# Field‐Cycling NMR Relaxometry in Tire Elastomer Science

**DOI:** 10.1002/mrc.70117

**Published:** 2026-05-24

**Authors:** Francesca Nardelli, Michele Pierigé, Elisa Carignani, Marco Geppi, Francesca Martini, Lucia Calucci

**Affiliations:** ^1^ Dipartimento di Chimica e Chimica Industriale Università di Pisa Pisa Italy; ^2^ Istituto di Chimica dei Composti OrganoMetallici ‐ ICCOM Consiglio Nazionale delle Ricerche ‐ CNR Pisa Italy

**Keywords:** ^1^H, cross‐linking, elastomers, field‐cycling, NMR, polymer dynamics, relaxometry, segmental dynamics

## Abstract

Field‐cycling (FC) NMR relaxometry is an ideal tool to investigate multiscale polymer dynamics since it can give access to longitudinal relaxation rates (*R*_1_(*ω*)) over a broad Larmor frequency range, which can be further extended by employing the frequency–temperature superposition principle. Applications of FC NMR on elastomers of interest for tire industry (i.e., *cis*‐1,4‐polyisoprene, *cis*‐1,4‐polybutadiene, and co‐poly [styrene‐butadiene]), aimed to obtain information on segmental and polymer dynamics over different regimes as a function of chain length in polymer melts and in dependence on cross‐linking degree and addition of fillers or additives in rubbers, are here reviewed for the first time. After a brief presentation of theoretical tools used in data analysis, studies on polymer melts at variable temperature are reported allowing theories for polymer dynamics, such as tube reptation and renormalized Rouse models, to be tested in the entanglement regime through characteristic dependences of *R*_1_(*ω*) on Larmor frequency. Then, studies on rubbers and elastomer compounds are illustrated, highlighting the effects of curing conditions (i.e., temperature, concentration of sulfur and accelerant), and the presence of reinforcing fillers and additives (e.g., tackifying resins) on segmental dynamics, related to macroscopic properties as glass transition temperature, and on collective polymer dynamics. Issues connected with the application of FC NMR relaxometry to polymeric systems with increasing structural and compositional complexity are critically discussed.

## Graphical abstract

Field‐cycling NMR relaxometry has established as an ideal tool to investigate multiscale polymer dynamics. Here, we review and discuss applications on elastomers and elastomer compounds of interest for the tire industry to obtain information on segmental dynamics and on polymer dynamics over different regimes as a function of chain length in polymer melts and in dependence on cross‐linking degree and addition of fillers and additives in rubbers.

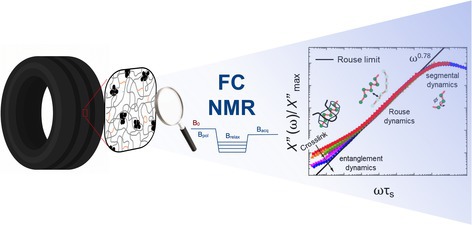

## 1 Introduction

Tires are mainly constituted by rubber, a material showing peculiar elasticity, resilience, and toughness properties, obtained by cross‐linking elastomers through curing. Elastomers (elastic polymers) are polymers with high elasticity that recover their original shape after being stretched significantly. This property is due to their long, irregularly coiled chains held together by weak intermolecular forces and physical entanglements that can spontaneously return to their compact random arrangement after being straightened by an applied force, thanks to the random motions of their repeating units through rotations around carbon–carbon bonds [1]. At ambient temperature, elastomers are amorphous materials above their glass transition temperature (*T*_g_), which makes them relatively soft and deformable [2, 3]. The term elastomer is often used interchangeably with rubber, although the latter is preferred when referring to vulcanized materials.

The elastomer with the longest history of use in tire rubber production is *cis*‐1,4‐polyisoprene (PI), the principal constituent of natural rubber (NR). NR is still an important material, but it now competes with synthetic polymers as *cis*‐1,4‐polybutadiene (PB) and co‐poly (styrene‐butadiene) (PSB), also referred to as butadiene rubber (BR) and styrene‐butadiene rubber (SBR), derived from by‐products of petroleum and natural gas [3].

**FIGURE 1:**
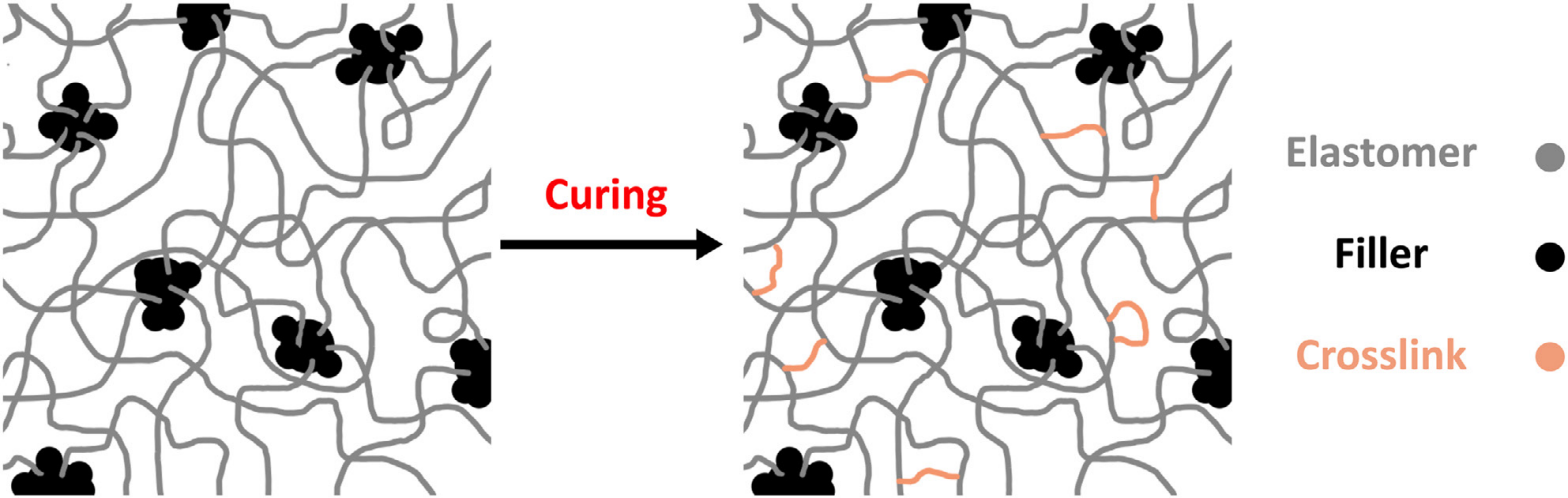
Sketch of uncured and cross‐linked elastomers' structure.

To obtain rubbers with desired viscoelastic and mechanical properties, elastomers are mixed with other ingredients and chemically cross‐linked by curing processes to form a tridimensional elastic network (Figure 1). The general compounding ingredients are broadly classified into six categories: polymers (natural or synthetic), cure package (sulfur, peroxide, metal oxide), fillers (reinforcing and nonreinforcing particles), age resistors (antioxidants and antiozonants), processing aids (plasticizers, oils, tackifiers), and miscellaneous (blowing agent, colorants, and flame retardants). In rubber formulation, ingredients concentration is generally indicated in phr (parts per hundred rubber).

The mechanical, viscoelastic, and thermal properties of elastomers, in particular the glass transition behavior, and their changes upon compounding and cross‐linking are strongly related to the microscopic dynamics of polymer chains [4, 5, 6, 7, 8, 9, 10, 11]. The investigation of elastomer chain dynamics is therefore of interest from a fundamental point of view and for establishing relationships with macroscopic properties that are important for the design of new rubber materials for specific applications.

Polymer chains in concentrated solutions and melts show dynamics over a wide frequency scale, from the fast scale of local segmental dynamics and internal motions (10^7^–10^12^ Hz) to the very slow scale of diffusive motions of the center of mass (1–10^3^ Hz). At the local scale, dynamics can be described in terms of the so‐called Kuhn segment, a mathematical construct representing an effective unit of chain length (or mass) that can adopt arbitrary orientations with respect to its neighboring units [12]. Fluctuations within this segment probe all conformations allowed by rotational isomerism of the chemical structure with mean correlation time *τ*_s_. These cooperative motions, also referred to as “glassy dynamics”, relate to the glass transition phenomenon, so that *τ*_s_ may be identified with the correlation time of the α‐process (*τ*_s_ = *τ*_α_). Segmental dynamics drives the polymer dynamics by determining the monomeric friction coefficient and is responsible for the non‐Arrhenius temperature dependence of correlation times in polymer melts. If mobile side‐groups exist in the polymer, their reorientations may be supplemented and superimposed to the main‐chain local segmental motions [13]. Slower polymer‐specific (Rouse and reptation) collective motions, also referred to as “polymer dynamics”, arise from the connection of Kuhn segments into chains. For linear polymers with molar mass *M* below the critical value *M*_e_ (average molar mass between entanglements), polymer dynamics is well‐described by the Rouse theory [14]. For polymers with *M* exceeding *M*_e_, physical entanglements occur, and the dynamics of a tagged chain is affected by topological constraints imposed by neighboring chains. Several models have been devised to describe the collective dynamics of entangled chains, including the tube reptation (TR) model developed by De Gennes [15] and Doi and Edwards [16], the n‐renormalized Rouse model [17, 18, 19, 20], and the mode‐mode coupling (MC) model [21, 22]. All the models predict characteristic power law dependences of the segmental mean‐squared displacement (MSD, $\left\langle r^{2}\left(t\right)\right\rangle$) on time, $\left\langle r^{2}\left(t\right)\right\rangle \propto t^{\alpha }$.

**FIGURE 2:**
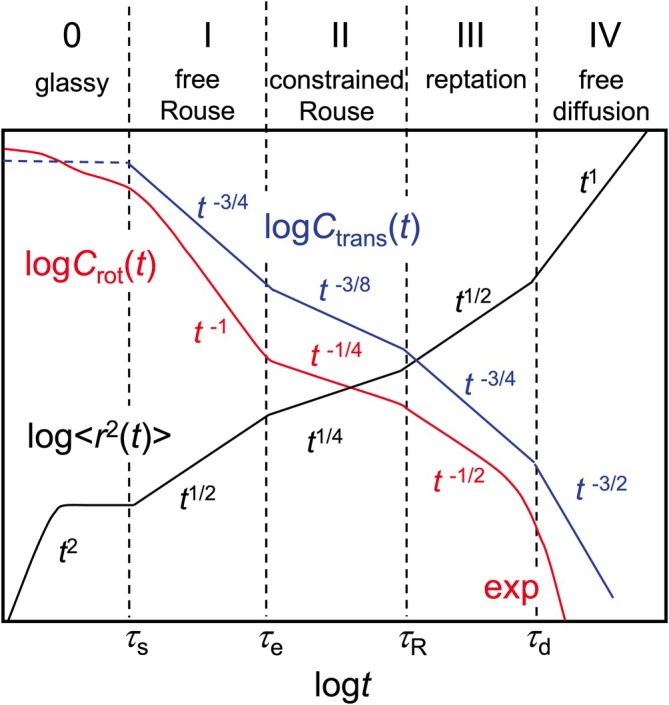
Dependences on time of mean squared displacement ( $\left\langle r^{2}\left(t\right)\right\rangle$) and of rotational (*C*_rot_(*t*)) and translational (*C*_trans_(*t*)) correlation functions according to the TR model.

In the most accepted TR model, the constraints imposed by the polymer matrix on a tagged chain are represented by a fictitious curved tube. Provided that the time scales for the different types of motions are well‐separated, four power law regimes (I–IV) are predicted for $\left\langle r^{2}\left(t\right)\right\rangle$ at progressively longer time and larger spatial scales, as shown in Figure 2: Regime I (*τ*_s_ < *t* ≤ *τ*_e_) is associated to free Rouse dynamics, not yet affected by entanglements; Regime II (*τ*_e_ < *t* ≤ *τ*_R_) includes constrained Rouse dynamics or local reptation in the tube; Regime III (*τ*_R_ < *t* ≤ *τ*_d_) reflects a subdiffusive process (reptation) along the tube which is terminated by the tube disengagement time *τ*_d_; Regime IV (*t* > *τ*_d_) consists of translational diffusion and isotropic reorientation of the entire polymer chain [23]. Regimes I–III are characterized by subdiffusive motions with *α* ≤ 0.5, whereas in the fourth regime molecules undergo Fickian diffusion (*α* = 1). To these regimes, segmental dynamics is added as regime 0.

Upon curing, segmental dynamics slows down due to the increase of the molecular friction coefficient, thus shifting the crossover times of polymer dynamics. Moreover, the formation of permanent cross‐links in the curing process of elastomers imposes further geometric constraints on chain collective dynamics, in addition to transient physical entanglements. Rubbers have therefore been considered as polymer melts with fixed chemical constrains. For entangled polymer networks, Lang and Sommer proposed a model in which a phantom network with cross‐links attached to an elastic background is considered, and segmental fluctuations of chain strands between cross‐links are confined within a tube determined by entanglements with neighboring chains [24, 25]. The chain segments slide along the tube, similarly to the case of polymer melts, but reaching a constant finite value of the MSD in the long‐time limit. Additional effects on chain dynamics of elastomers may arise from interactions with additives such as filler particles, resins, and oils.

Polymer dynamics is usually investigated by rheological [26] and dielectric relaxation techniques [27, 28, 29, 30], neutron scattering [31, 32], and NMR techniques. In particular, several time‐domain NMR methods [33, 34, 35, 36], including free induction decay (FID) analysis, Carr–Purcell–Meiboom–Gill (CPMG) experiments, multiquantum experiments, dipolar correlation effect, Hahn and solid echoes, longitudinal relaxation times measurements, and field gradient (FG) NMR experiments, have been exploited to probe dynamics of elastomers over different time scales.

**FIGURE 3:**
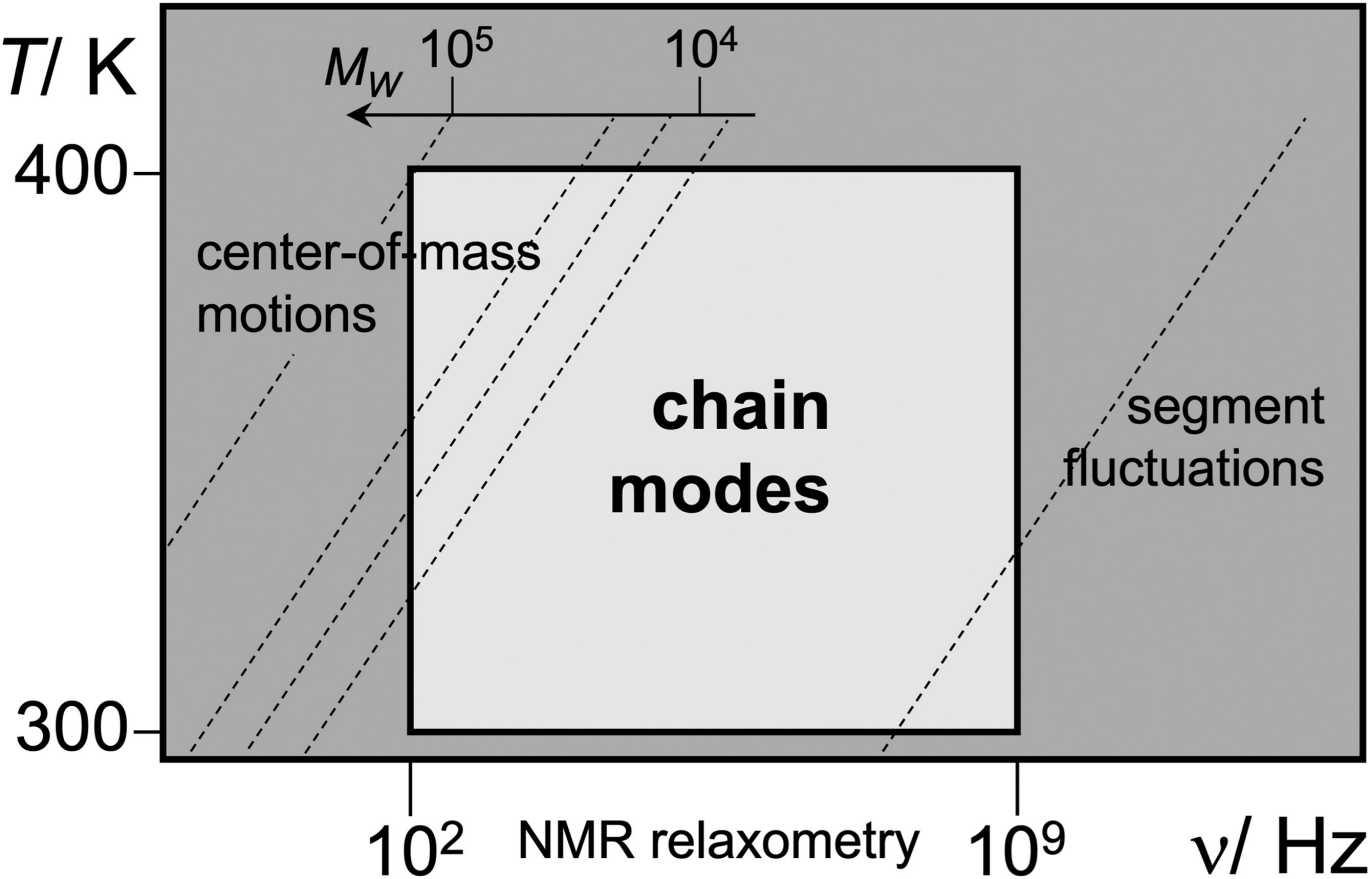
Schematic representation of the experimental temperature/frequency window conveniently accessible by FC NMR relaxometry in combination with conventional high‐field NMR techniques. Adapted from Kimmich and Fatkullin [13].

Among them, since the pioneering work of Kimmich and coworkers [13], ^1^H field‐cycling NMR (FC NMR) relaxometry has been established as a powerful technique for the investigation of the multiscale dynamics of polymers over a wide and favorable range of observable frequencies (10^3^–10^10^ Hz, Figure 3) [37, 38, 39, 40, 41, 42, 43, 44]. In fact, ^1^H FC NMR measures the dependence on Larmor frequency (*ν* or *ω* = 2π*ν*) of the proton spin–lattice relaxation rate, *R*_1_(*ω*) = 1/*T*_1_(*ω*), also referred to as nuclear magnetic relaxation dispersion (NMRD). Commercial FC NMR relaxometers, available since the end of the 90s, cover a frequency range of 10 kHz to 40 MHz for ^1^H [45]. By employing Earth's field compensation, frequencies down to some 100 Hz have been reached with home‐made relaxometers [46, 47, 48, 49, 50]. Recently, experiments at frequencies as low as 3 Hz have been reported [51]. Moreover, the frequency range can be extended to higher frequencies (100 MHz—1 GHz) by using conventional NMR spectrometers.

In the case of ^1^H nuclei, spin–lattice relaxation is determined by fluctuations of the proton spins due to reorientational and translational dynamics of polymer segments. As better explained in Section 2, both intramolecular and intermolecular relaxation pathways contribute to *R*_1_(*ω*), the first being determined by reorientational motions, which dominate at high frequencies, the latter being also affected by translational motions, especially at low frequencies. The *R*_1_(*ω*) dispersion reflects the spectrum of motions of ^1^H‐^1^H spin pairs and can be expressed as a linear combination of spectral densities, *J*(*ω*), the Fourier transform of the dipolar autocorrelation functions, *C*(*t*). Therefore, the NMRD curves may be analyzed to get information on both segmental and collective polymer dynamics.

^1^H FC NMR relaxometry has been indeed extensively applied to study the dynamics as a function of temperature in the molten state of linear polymers with molar mass both below and above the entanglement limit, with the aim of testing models of polymer dynamics through the dependence of *R*_1_ on Larmor frequency (*R*_1_(*ω*)∝*ω* ^‐*γ*^), reflecting the dependence of *C*(*t*) on time (*C*(*t*)∝*t* ^*−α*^) predicted by different models [12, 41, 43, 49, 52, 53, 54, 55, 56, 57, 58, 59, 60]. ^1^H FC NMR relaxometry data were firstly reviewed by Kimmich and Fatkullin [13], who interpreted the apparent universal dispersion behavior of *R*_1_(*ω*) at *ωτ*_s_ ≪ 1, with characteristic power law regimes, within the Rouse and renormalized Rouse theories for nonentangled and entangled polymers, respectively. Lately, Rössler and co‐authors [52, 53, 54, 56, 59, 60] reinvestigated the ^1^H *R*_1_ dispersion behavior of several elastomers, adding new data and proposing different procedures for data analysis. In particular, the *R*_1_(*ω*) data are transformed into the susceptibility representation $\chi^{\prime \prime }\left(\omega \right)=\omega R_{1}\left(\omega \right)$ and master curves of $\chi^{\prime \prime }\left(\omega \tau_{s}\right)$ are built (see Section 2) on the basis of the frequency–temperature superposition (FTS) principle. This principle assumes that the spectral shape of the relaxation response remains essentially unchanged with temperature, while only the characteristic timescale is shifted. This implies that different relaxation processes share the same temperature dependence. This condition typically applies for polymers at temperatures well above *T*_g_, which is indeed the case for tire elastomers at room and higher temperatures. Under these conditions, NMR susceptibility curves measured at different temperatures can be shifted and combined to extend the accessible frequency range [26, 61]. Using this procedure, contributions from segmental and polymer collective dynamics have been separated and *τ*_s_ values have been determined as a function of temperature.

A much smaller number of papers have been reported in the literature concerning the use of ^1^H FC NMR relaxometry to investigate the effects on elastomers dynamics of cross‐linking and curing parameters (i.e., temperature, sulfur content, and sulfur/accelerator ratio), as well as addition of fillers and additives [23, 62, 63, 64, 65, 66, 67, 68, 69, 70, 71, 72, 73].

In this review, the applications of ^1^H FC NMR relaxometry to the investigation of elastomer dynamics will be summarized and critically discussed. After a brief recapitulation of theoretical tools (Section 2 and Appendix A [Table A1]), studies of dynamics of pristine elastomers in the molten state, mostly dedicated to the check of theories for polymer dynamics in the entanglement regime and to the set‐up of tools for the analysis of FC NMR data to extract information on both segmental and polymer dynamics, will be presented (Section 3). In the following sections, the effects on dynamics of vulcanization (Section 4) and addition of fillers and resins to elastomer compounds (Section 5) will be illustrated.

## 2 Theoretical Background

For protons, the main interaction controlling spin–lattice relaxation is the magnetic dipole–dipole interaction between different ^1^H‐^1^H spin pairs in the sample [74]. The interaction Hamiltonian results from the sum over all pairs of spins i,j separated by vectors ***r***_ij_. For each pair, the interaction term depends on the orientation of ***r***_ij_ with respect to the magnetic field, as well as on the distance *r*_ij_. In the case of polymers, the summation is performed over both spins belonging to the same segment, which constitutes the intramolecular contribution, and spins from different segments or chains, giving the intermolecular contribution. The modulation of the dipole–dipole interactions by motions results in two different contributions to *R*_1_: *R*_1,*intra*_ arising from reorientations dominates at high frequency and *R*_1,*inter*_, arising from both reorientational and translational dynamics, becomes increasingly important at low frequencies [12, 44, 57, 58].

According to the Bloembergen–Purcell–Pound (BPP) theory [75], relaxation rates are connected to spectral densities through the equation

(1) $$R_{1,i}\left(\omega \right)=K_{i}\left[J_{i}\left(\omega \right)+4J_{i}\left(2\omega \right)\right]$$

where *K*_*i*_ is a proportionality constant depending on the second moment of the relevant intrachain or interchain dipolar interactions.

The spectral density for segmental dynamics has been phenomenologically described by the Cole–Davidson (CD) function: [27]

(2) $$J_{\mathrm{CD}}\left(\omega \right)=\frac{\sin\left[\beta_{\mathrm{CD}}\arctan\left(\omega \tau_{\mathrm{CD}}\right)\right]}{\omega {\left[1+{\left(\omega \tau_{\mathrm{CD}}\right)}^{2}\right]}^{\frac{\beta_{\mathrm{CD}}}{2}}}$$

where 0 < *β*_*CD*_ ≤ 1 and the characteristic correlation time *τ*_*CD*_ is related to *τ*_s_ by the expression *τ*_s_ = *β*_*CD*_ *τ*_*CD*_.

For collective polymer dynamics at *t* ≫ *τ*_s_, dipolar rotational (*C*_*rot*_(*t*)) and translational (*C*_*trans*_(*t*)) autocorrelation functions proportional to power laws of $\left\langle r^{2}\left(t\right)\right\rangle$ can be written, which result in different power dependences on time [40, 41, 57, 58, 76]. In particular, $C_{\text{trans}}\left(t\right)\propto {\left\langle r^{2}\left(t\right)\right\rangle }^{-\frac{3}{2}}$, whereas in the case of *C*_*rot*_(*t*), there is no universal relationship on MSD, but it depends on the coupling assumed between rotation and translation in a given model.

The different scaling relations predicted by the various models reflect the fundamentally different way in which entanglement effects imposed by surrounding chains are treated. In the so‐called “isotropic” models, such as renormalized Rouse and MC models, the influence of neighboring chains is incorporated in an averaged, dynamical manner through a memory function representing the time correlation of intermolecular forces. As a consequence, segment movements progressively lose memory of the initial chain conformation, leading to effectively isotropic displacements in the absence of geometrical confinement. In contrast, in the TR model, topological constraints arising from the surrounding chain confine segmental displacements within a fictitious tube‐like region and preserve correlations with the initial conformation over longer times. Accordingly, in isotropic models, the segmental displacements are considered uncorrelated with the initial conformation of the polymer chain already at *t* > *τ*_s_, and *C*_*rot*_(*t*) depends on $\left\langle r^{2}\left(t\right)\right\rangle$ according to the equation:

(3) $$C_{\mathrm{rot}}^{\mathrm{iso}}\left(t\right)\propto \frac{1}{{\left\langle r^{2}\left(t\right)\right\rangle }^{2}}$$

In contrast, in the TR model, segmental displacements at times *τ*_s_ < *t* < *τ*_d_ are restricted within the tube so that conformations remain strongly correlated with the initial one. In this case,

(4) $$C_{\mathrm{rot}}^{\mathrm{TR}}\left(t\right)\propto \frac{1}{\left\langle r^{2}\left(t\right)\right\rangle }$$

These differences directly translate into distinct power law exponents in the frequency dependence of *R*_1_(*ω*), which can be used to discriminate between the two models in FC NMR experiments. The dependence of *C*_*rot*_(*t*) on time in the different regimes of polymer dynamics is reported in Table 1 together with the corresponding dependence of *R*_1,*intra*_ on frequency for the different models [57, 58, 77, 78]; the dependences on time of C_*trans*_(*t*) and C_*rot*_(*t*) for the TR model are shown in Figure 2.

**TABLE 1:** Theoretical expressions of the rotational second‐rank autocorrelation function (*C*_*rot*_(*t*)) and of the corresponding intramolecular *R*₁(*ω*) and $\chi^{\prime \prime }\left(\omega \right)$ contributions in the different regimes of polymer dynamics as derived from the tube–reptation [15, 16] and first renormalized Rouse models [17, 18, 19, 20].

Tube–Reptation model				
Dynamic regime	Limit	*C*_*rot*_(t)	$R_{1}\left(\omega \right)\propto \omega^{-\gamma }$	$\chi^{\prime \prime }\left(\omega \right)\propto \omega^{1-\gamma }$
Rouse^a^ (Regime I)	*τ*_s_ < *t* ≤ *τ*_e_	∝ *t* ^*−*1^	∝ ln*ω*	∝ *ω*ln*ω*
Constrained Rouse (Regime II)	*τ*_e_ < *t* ≤ *τ*_R_	∝ *t* ^−0.25^	∝ *ω* ^*−*0.75^	∝ *ω* ^0.25^
Reptation (Regime III)	*τ*_R_ < *t* ≤ *τ*_d_	∝ *M* ^0.5^ *t* ^−0.5^	∝ *ω* ^*−*0.5^	∝ *ω* ^0.5^
Free diffusion (Regime IV)	*t* > *τ*_d_	*e* ^−2*t*^	∝ *ω* ^0^	∝ *ω* ^1^

Renormalized Rouse model				
“High‐mode number limit”	*τ*_s_ < *t* ≤ *τ*_e_	∝ *t* ^−0.5^	*ω* ^*−*0.5^	*ω* ^0.5^
“Low‐mode number limit”	*τ*_e_ < *t*	∝ *t* ^−0.8^	*ω* ^*−*0.8^	*ω* ^0.2^

^a^ In the Rouse regime, the dependence of *R*_1_ on *ω* is logarithmic, but in a double logarithmic plot, the *R*_1_ dispersion appears approximately linear with a slope *γ* ≃ 0.25 (1–*γ* ≃ 0.75 in the $\chi^{\prime \prime }$ representation).

According to the fluctuation–dissipation theorem, the spectral density of thermal equilibrium fluctuations of a property is related to the linear response of that property to a weak external perturbation, that is, to the susceptibility function. The imaginary part of the susceptibility is expressed as $\chi^{\prime \prime }\left(\omega \right)=\mathrm{\omega J}\left(\omega \right)$. This relationship can be exploited to convert FC NMR data to the susceptibility representation, where one can write the following: [49, 52, 54, 78, 79]

(5) $$\omega R_{1}\left(\omega \right)=K\left[\chi^{\prime \prime }\left(\omega \right)+2\chi^{\prime \prime }\left(2\omega \right)\right]$$

This expression can be approximated to $\omega R_{1}\left(\omega \right)≅3K{\tilde{\chi }}^{\prime \prime }\left(\omega \right)$, where ${\tilde{\chi }}^{\prime \prime }\left(\omega \right)$ is the “normalized NMR susceptibility” and 3 is a normalization factor to provide an integral over ${\tilde{\chi }}^{\prime \prime }\left(\omega \right)$ of π/2. Moreover, a (nonnormalized) total FC NMR susceptibility $\chi_{\text{DD}}^{\prime \prime }\left(\omega \right)=\omega R_{1}\left(\omega \right)$ can be introduced to analyze FC NMR data, especially in comparisons with other techniques for the investigation of dynamics that give access to susceptibility, such as dielectric spectroscopy and rheology [58].

According to the FTS principle, all relaxation processes contributing to the polymer dynamics are assumed to share the same temperature dependence as *τ*_s_(*T*), without changing their spectral shapes. Therefore, a susceptibility master curve covering local segmental as well as collective Rouse and entanglement dynamics can be built by shifting $\chi^{\prime \prime }\left(\omega \right)$ curves collected at different temperatures along the frequency axis until they overlap. The master curve can be expressed as a function of frequency reduced by the correlation time for segmental dynamics, as $\chi^{\prime \prime }\left(\omega \tau_{s}\right)$. Under the assumption that *K* does not change significantly with temperature, master curves can also be obtained for $\chi_{\text{DD}}^{\prime \prime }\left(\omega \right)$, in the following simply indicated as $\chi^{\prime \prime }\left(\omega \right)$. Such master curves typically cover 10 decades in frequency and, when terminal dynamics is reached, allow transformation into the time domain yielding the corresponding correlation function. Master curves of the spectral density, corresponding to $R_{1}\left(\omega \tau_{s}\right)/\tau_{s}$, can also be obtained by dividing $\chi^{\prime \prime }\left(\omega \tau_{s}\right)$ by *ωτ*_s_.

Considering that polymer and glassy dynamics are statistically independent and well‐separated in time, $\chi^{\prime \prime }\left(\omega \right)$ can be expressed as

(6) $$\chi^{\prime \prime }\left(\omega \right)=\left(1-f\right)\chi_{\text{glass}}^{\prime \prime }\left(\omega \right)+f\chi_{\text{pol}}^{\prime \prime }\left(\omega \right)$$

where *f* represents the fractional contribution of polymer dynamics to the total spectrum of motions. The relation $S=\sqrt{f}$ connects *f* to the dynamic order parameter *S*, which represents a measure of the spatial restrictions imposed on segmental motions by chain connectivity and entanglements. However, being derived from a ^1^H‐^1^H dipolar autocorrelation function, *S* also depends on the angles (*θ*_ij_) between the internuclear vectors of ^1^H‐^1^H spin pairs and the contour of the polymer chain:

(7) $$S=\frac{1}{2}\left(3\cos^{2}\theta_{\mathrm{ij}}-1\right)S_{\text{chain}}$$

where *S*_*chain*_ is the chain order parameter.

As firstly pointed out by Kimmich and Fatkullin [13], the total spectrum of motions of a polymer is dominated by local segmental dynamics. As a consequence, $\chi_{\mathrm{pol}}^{\prime \prime }\left(\omega \right)$ can be correctly interpreted only after subtracting $\chi_{\text{glass}}^{\prime \prime }\left(\omega \right)$ from $\chi^{\prime \prime }\left(\omega \right)$. $\chi_{\text{glass}}^{\prime \prime }\left(\omega \right)$ can be determined experimentally as the total $\chi^{\prime \prime }\left(\omega \right)$ for polymers with very small molar mass, which do not undergo Rouse or entangled polymer dynamics. Furthermore, it can be theoretically expressed, using the CD spectral density (Equation (2)), as

(8) $$\chi_{\text{glass}}^{\prime \prime }\left(\omega \right)=\omega K_{\mathrm{CD}}\left[J_{\mathrm{CD}}\left(\omega \right)+4J_{\mathrm{CD}}\left(2\omega \right)\right]$$

Since $\chi_{\text{glass}}^{\prime \prime }\left(\omega \right)$ coincides with $\chi^{\prime \prime }\left(\omega \right)$ for frequencies larger than ≈1/*τ*_s_, *τ*_s_ can be determined by fitting the high‐frequency branch of the $\chi^{\prime \prime }\left(\omega \right)$ curves, ascribable to the sole glassy dynamics, to Equation (8).

*τ*_s_ correlation times determined for elastomers usually exhibit non‐Arrhenius temperature dependence, as expected for the α‐relaxation process. The Vogel–Fulcher–Tammann (VFT) empirical equation [26]

(9) $$\tau_{s}=\tau_{0}\exp\left(\frac{DT_{0}}{T-T_{0}}\right)$$

is generally used to describe *τ*_s_ trends, where *τ*_0_ is a pre‐exponential factor, *T*_0_ is the temperature at which the correlation time diverges to infinity, and *D* is a fragility factor. The glass transition temperature, *T*_g_, has been established as the temperature at which *τ*_s_ = 100 s [80]. A measure of polymer fragility, that is of the rapidity with which glassy dynamics slows down on cooling the polymer toward the glass transition, can be given by determining the fragility index *m* defined as

(10) $$m=-{\left.\left(\frac{\text{dLog}\tau_{s}\left(T/T_{g}\right)}{d\left(T/T_{g}\right)}\right)\right|}_{T_{g}}$$

Blochowicz et al. [81] recast the VFT equation in terms of *m* and *T*_g_ to obtain the following:

(11) $$\mathrm{Log}\left[\frac{\tau_{s}\left(T\right)}{\tau_{0}}\right]=\frac{\mathrm{Lo}g^{2}\left[\frac{\tau_{s}\left(T_{g}\right)}{\tau_{0}}\right]}{m\left(\frac{T}{T_{g}}-1\right)+\mathrm{Log}\left[\frac{\tau_{s}\left(T_{g}\right)}{\tau_{0}}\right]}$$

Based on this equation, polymers with the same *τ*_0_ and *m* show a superposition of Log (*τ*_s_) vs *T*/*T*_g_‐1 curves.

## 3 Dynamics in Linear Polymer Melts

In the last decades, ^1^H FC NMR relaxometry has been successfully applied to the study of linear long polymers at temperatures far above *T*_g_ (polymer melts), as an experimental approach to test theories of polymer dynamics. Many different polymers have been investigated including PB and PI, of interest for the tire industry [40, 49, 50, 52, 53, 54, 56, 79, 82, 83, 84, 85]. All studies report monoexponential recovery curves of longitudinal magnetization on time in the measurement of *R*_1_(*ω*) in ^1^H FC NMR experiments. This can be accounted for by considering that chemically inequivalent protons are separated by relatively short distances, so that their longitudinal relaxation times are averaged by spin diffusion, and that molecular weight polydispersity, presence of chain end groups with enhanced dynamics, and sample heterogeneity are not sufficient to give rise to a broad distribution of relaxation times.

**FIGURE 4:**
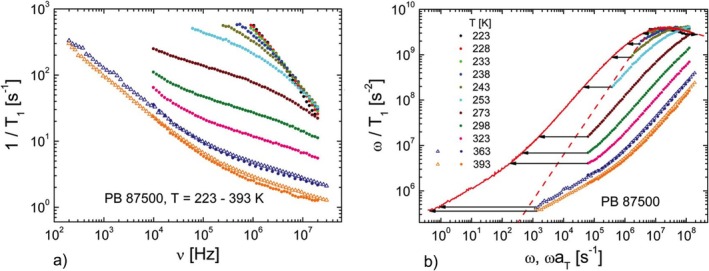
(a) Dispersion of $R_{1}\left(\omega \right)=1/T_{1}\left(\omega \right)$ for PB with *M* = 87,500 g mol^−1^ in the indicated temperature range measured with (open triangles) and without (full circles) compensation for stray fields. (b) Susceptibility representation, $\chi^{\prime \prime }\left(\omega \right)=\omega /T_{1}\left(\omega \right),$ of the same data as in (a). At the lowest temperatures, the peak associated to segmental dynamics is discernible and fitted with a Cole–Davidson function (dashed red line). Arrows illustrate frequency−temperature superposition, which is applied to create a master curve (solid red line). The color for temperatures is equivalent in both panels. Reprinted with permission from Herrmann et al. [49] Copyright 2012 (ACS).

The ^1^H NMRD curves of highly entangled polymers revealed distinct power law dependences on frequency, which reflect the different regimes of chain dynamics, and were compared with the theoretical expressions of *R*_1_(*ω*) derived from the TR and renormalized Rouse models (Table 1). Figure 4a shows the ^1^H NMRD curves of PB with *M* = 87,500 g mol^−1^ at variable temperatures in the 223‐293 K range, collected by Herrmann et al. [49] The dependence of *R*_1_ on the Larmor frequency varies systematically with temperature, reflecting the progressive acceleration of motions on heating and the corresponding shift of the dynamic regimes accessible within the experimental frequency window. Three frequency regions, characterized by distinct slopes arising from the *R*_1_(*ω*) ∝ *ω* ^−γ^ dependences, can be identified. In the earliest works [52, 53, 54, 55, 79, 82], the interpretation of these regions was debated, and they were alternatively explained with the classical Doi–Edwards TR model or the n‐renormalized Rouse model. However, more recent studies [40, 49, 50, 56, 84], also supported by comparisons with double‐quantum (DQ) NMR [86, 87], FG NMR [83, 84], neutron scattering [40, 50], and FC NMR investigations at extremely low fields [49], demonstrated that the observed ^1^H *R*_1_(*ω*) trends can be consistently explained in the framework of the TR model, which will be the reference model in the following discussion. The regions observed in the *R*_1_(*ω*) dispersions, from low temperature and high frequency to high temperature and low frequency, are as follows: (i) a region with *γ* ≈ 1, associated with the local segmental motions related to the glass transition; (ii) a region with *γ* ≈ 0.25, ascribed to regime I (Rouse dynamics); and (iii) a region with *γ* ≈ 0.68, corresponding to regime II (constrained Rouse dynamics).

The crossover frequencies between these regimes shift progressively to higher values as the temperature increases, indicating faster molecular motions. At low temperatures (*T* ≤ 238 K), the relaxation behavior is dominated by local segmental dynamics associated with the glass transition (*T*_g_∽175 K). Upon heating, an additional regime emerges at lower frequencies, reflecting the onset of Rouse dynamics. At the highest temperatures (*T* ≥ 323 K), the low‐frequency side becomes dominated by the constrained Rouse regime. It should be noted that, due to the still limited accessible Larmor frequency window at the low‐frequency end, slow subdiffusive (reptation, Regime III) and diffusive motions of high‐molecular weight entangled polymers cannot be probed by FC NMR, as their characteristic correlation times are too long. These slow dynamic processes have instead been successfully investigated by complementary NMR techniques, including DQ NMR [86, 87] and FG NMR [36, 88].

The NMR susceptibility spectra derived from the longitudinal relaxation data are reported in Figure 4b. At low temperatures (*T* ≤ 238 K), where segmental dynamics dominates the relaxation behavior, the $\chi^{\prime \prime }\left(\omega \right)$ spectra display a maximum corresponding to the condition *ωτ*_s_ ≃ 0.6. As the temperature increases, $\chi^{\prime \prime }\left(\omega \right)\propto \omega^{1-\gamma }$ power law dependences characteristic of the Rouse and constrained Rouse regimes progressively emerge at intermediate and low frequencies. In the susceptibility representation, a $\chi^{\prime \prime }\left(\omega \tau_{s}\right)$ master curve (solid red line in Figure 4b) can be built from the spectra at different temperatures by applying the FTS principle [49, 53, 54, 55, 79], as described in Section 2.

**FIGURE 5:**
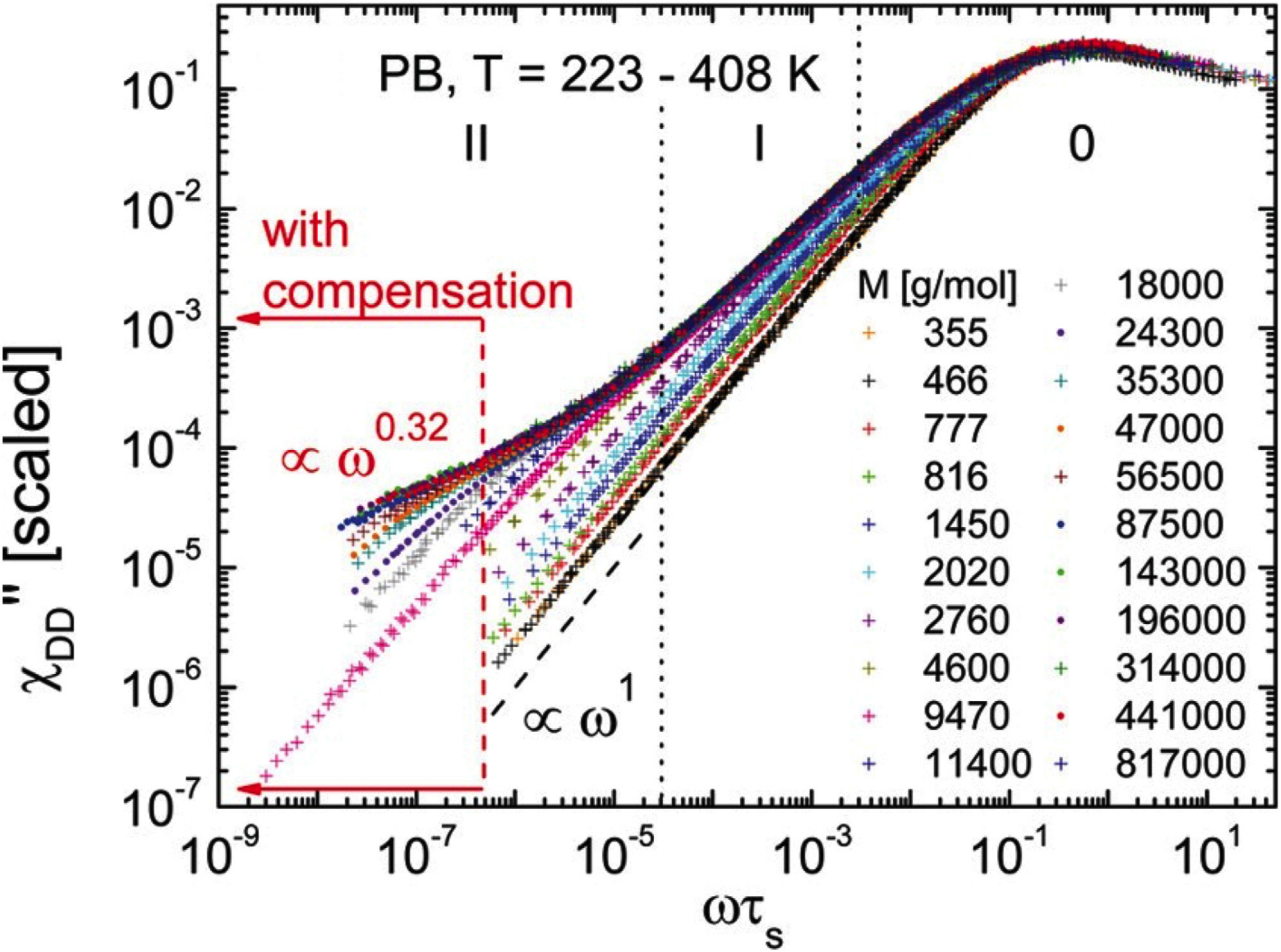
Susceptibility master curves as a function of the reduced frequency *ωτ*_s_ ( $\chi^{\prime \prime }\left(\omega \tau_{s}\right)$) built from longitudinal relaxation data on PB samples with the indicated *M* values in the 223–408 K temperature range. The frequency range in which *R*_1_ has been measured while employing the compensation for stray fields is marked by arrows. Relaxation regimes 0, I, and II, that is, glassy dynamics, Rouse and entanglement dynamics, respectively, are separated by vertical dotted lines. Reprinted with permission from Herrmann et al. [49] Copyright 2012 (ACS).

Master curves of $\chi^{\prime \prime }\left(\omega \tau_{s}\right)$ were constructed for different polymers, including PB, PI, and PSB, of various molecular weights, from oligomers (*M* < *M*_e_) to highly entangled polymers (*M* ≫ *M*_e_) [49, 53, 54, 56, 79]. For all polymers investigated so far, a qualitatively similar evolution of the NMR susceptibility spectra with increasing molecular weight has been observed. As an example, $\chi^{\prime \prime }\left(\omega \tau_{s}\right)$ spectra are shown in Figure 5 for a series of PB samples with molecular weights ranging from 355 to 817,000 g mol^−1^. In the low‐*M* limit (*M* = 355 and 466 g mol^−1^), the $\chi^{\prime \prime }\left(\omega \tau_{s}\right)$ spectra correspond to those of a simple glass‐forming liquid, where only segmental relaxation (regime 0) occurs. In this case, $\chi^{\prime \prime }\left(\omega \tau_{s}\right)$ exhibits a maximum in the high‐frequency branch (*ωτ*_s_  ≈ 0.6) and a linear (*ω* ^1^) dependence at low frequencies. By increasing *M*, the high‐frequency region of the spectra remains unchanged, indicating that segmental dynamics is independent of *M*. Conversely, in the low‐frequency region, features characteristic of polymer dynamics (regimes I and II) gradually emerge. In the frequency range 3 × 10^−5^ < *ωτ*_s_ < 3 × 10^−3^, $\chi^{\prime \prime }\left(\omega \tau_{s}\right)$ progressively increases due to the activation of additional Rouse modes, until it saturates at *M* ≈ 4600 g mol^−1^ ≈ 2 *M*_e_, where Rouse dynamics becomes fully established and remains unchanged upon further increasing *M*. For even higher molecular weights, features associated with entanglement dynamics start to appear at *ωτ*_s_ < 3 × 10^−5^, where a second power law dependence, $\chi^{\prime \prime }\left(\omega \right)\propto \omega^{1-\gamma \left(M\right)}$, becomes visible. The corresponding exponent, 1 − *γ*(*M*), progressively decreases with increasing *M*, approaching the theoretical value of 0.25. This behavior indicates a very gradual crossover toward the tube–reptation regime [49, 54, 56], which is not yet fully developed even at *M* ≈ 300 *M*_e_. For *M* ≤ 11,400 g mol^−1^, a further crossover to a Debye‐like power law (*ω* ^1^) is observed, which shifts to lower frequencies as *M* increases. This region corresponds to the terminal relaxation, associated with the diffusive motion of polymer chains upon disentanglement. At larger *M* values, this relaxation mode shifts progressively beyond the frequency window accessible by FC NMR measurements. The protracted transition toward full reptation and the higher‐than‐expected exponent in the constrained Rouse regime remain subjects of discussion. Possible explanations include weak deviations from the TR model, such as an early onset of terminal relaxation [49] and, at very high molecular weights, the contribution of additional relaxation mechanisms that are accounted for in refined versions of the Doi–Edwards theory, namely, contour length fluctuations (CLFs) [89, 90] and constraint release (CR) models [91, 92].

In the high‐molecular weight limit, all polymers display similar NMR susceptibility spectra, where distinct regions characterized by different power law dependences, corresponding to regimes 0, I, and II of segmental and polymer dynamics, can be identified (Figure 5). However, the relaxation behavior at low frequencies, where polymer‐specific contributions become significant, is not universal [55, 66]. Table 2 summarizes the exponents obtained from the $R_{1}\left(\omega \right)$ dispersion curves for the main polymers used in the tire industry, in the Rouse (I), and constrained Rouse (II) regimes.

**TABLE 2:** Exponents describing the power law dependences $R_{1}\left(\omega \right)\propto \omega^{-\gamma }$ in regimes I (Rouse) and II (constrained Rouse) of polymer dynamics obtained from experimental ^1^H NMRD curves of PB, PI, and PSB polymers with high molecular weight.

	*γ* exponent	
	Regime I	Regime II
PB	0.23 [55], 0.22 [66]	0.68 [55], 0.65 [66]
PI	0.17 [55], 0.15 [66]	0.35^a^ [55], 0.37^a^ [66]
PSB	0.25 [66]	0.48^a^ [66]

^a^ This value is only indicative, as the regime II dispersion was not sampled over a sufficiently wide low‐frequency range due to instrumental limitations, and only its onset was observed.

Different power law dependences were observed in the Rouse regime for the various polymers [55, 66]. For PB, *γ* = 0.22–0.23 was obtained, in good agreement with theoretical predictions (*γ* = 0.25, see Table 1). In contrast, for PI, the relaxation data in the same frequency region yielded an apparent exponent *γ* = 0.15–0.17. This deviation from the theoretically predicted behavior can be explained by the partial overlap between the contributions of segmental and polymer dynamics, which hinders an accurate determination of the slope in the Rouse regime. The relative weight of segmental and polymer dynamics in the NMR susceptibility spectra strongly depends on the molecular level structural features, leading to different values of the polymer fraction *f* (Equation (6)). In PB, proton pairs oriented nearly along the polymer backbone form a small angle with the chain contour, resulting in less efficient averaging of dipolar interactions corresponding to a high *S* value (Equation (7)). In contrast, in PI, where the internuclear vectors form larger angles relative to the chain contour, segmental motions average the dipolar interactions more efficiently resulting in a lower *S* value. This leads to a more pronounced contribution of segmental dynamics, compared to polymer dynamics, in the NMR susceptibility spectra and *R*_1_ dispersions. Herrmann et al. [55] demonstrated that universal polymer spectra, showing the theoretical exponents predicted for the Rouse regime, can only be obtained after subtracting the “spectrum of segmental dynamics” $\chi_{\text{glass}}^{\prime \prime }\left(\omega \right)$ from the total susceptibility $\chi^{\prime \prime }\left(\omega \right)$ (see Section 2).

Concerning the constrained Rouse regime observable in the low‐frequency region, the exponents (*γ* ≈ 0.35–0.68) obtained from NMRD curves are systematically lower than those predicted by the TR model (*γ* = 0.75, see Table 1). The observed deviation may be attributed to a very gradual (protracted) transition toward fully developed tube–reptation dynamics [49, 54, 56], established only at very high molecular weights, and/or to slight deviations from the theoretical model. However, caution must be exercised when interpreting the low‐frequency region of the NMRD curves, since intermolecular ^1^H‐^1^H dipolar couplings may no longer be negligible, as demonstrated by isotopic dilution studies performed on partially deuterated samples [50, 76, 93, 94]. The presence of intermolecular relaxation contributions could result in apparent power law exponents that do not accurately reflect the behavior of the second‐rank reorientational correlation function. Moreover, it should be noted that this exponent can be determined accurately only when the constrained Rouse regime spans a sufficiently broad frequency window. Even at the highest temperatures, this condition is fulfilled only if the experimental Larmor frequency range is extended to very low frequencies, well below the 10 kHz lower limit of commercial FC NMR relaxometers [49].

Besides testing models of polymer dynamics, FC NMR studies also enabled the determination of the correlation times of segmental dynamics, *τ*_s_, as a function of temperature. *τ*_s_ values were determined from the build‐up of the NMR susceptibility master curves, after establishing a reference value at low temperature, where a distinct maximum in $\chi^{\prime \prime }\left(\omega \right)$ is observed, through different methods. In some cases, the reference *τ*_s_ value was determined by fitting the low‐frequency flank of the susceptibility spectrum with a function describing the segmental dynamics contribution, typically a Cole–Davidson susceptibility function (Equation (8)). For PB, PI, and PSB, *β*_*CD*_ values of 0.25, 0.30, and 0.12 were found, respectively [66]. In other cases, the $\chi^{\prime \prime }\left(\omega \right)$ spectrum of a low‐molecular weight glass former or homologous oligomer was used as a reference to extract the value of *τ*_s_ at low temperature, given the independence of segmental dynamics from molecular weight [53, 54, 55, 79]. Sometimes, the condition *ωτ*_s_ ≃ 0.6 at the maximum was applied [69]. The values of *τ*_s_ at the other temperatures were obtained from the shift factors along the frequency axis used to overlap the corresponding $\chi^{\prime \prime }\left(\omega \right)$ curves to the reference one (Figure 4b).

**FIGURE 6:**
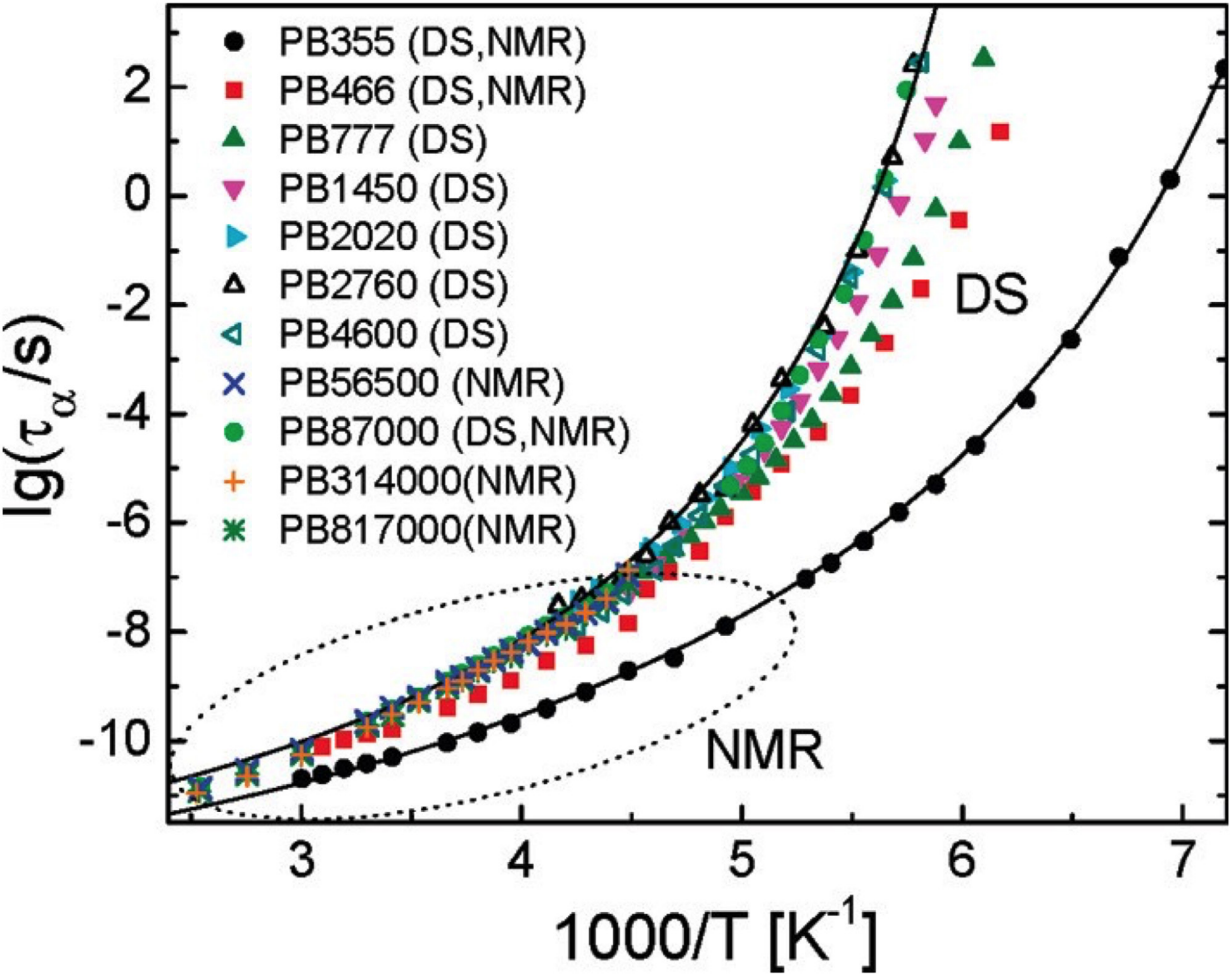
Time constants of the α‐process (segmental relaxation) of PB samples with different molecular weight as obtained by analyzing the dielectric spectra (DS) and the FC NMR spectra (the latter marked with ellipse); solid lines: interpolations with the Vogel–Fulcher–Tammann equation. Reprinted with permission from Kariyo et al. [79] Copyright 2008 (ACS).

Kariyo et al. [79] compared the values of *τ*_s_ obtained from the construction of $\chi^{\prime \prime }\left(\omega \tau_{s}\right)$ master curves for a series of PB samples with different molecular weights with the values of the structural α‐relaxation time values, *τ*_α_, simultaneously measured from dielectric spectra (Figure 6). The two sets of data were found to be in good agreement, confirming the analogy of the two quantities measured from these two independent techniques. The trends with temperature showed a non‐Arrhenius behavior typical of glass‐forming systems, which could instead be interpolated using the VFT equation (Equation (9)).

## 4 Effects of Cross‐Linking on Dynamics

The vulcanization of elastomers is a process discovered by Charles Goodyear in 1839, which largely expanded the application of rubbers to various fields, such as the tire industry, by conferring to these materials several desired macroscopic properties. In vulcanization, sulfur interlinks (‐S_*n*_‐) are created between chains by heating the polymer in the presence of sulfur and other ingredients (accelerator, ZnO, and stearic acid) that constitute the cure package. The formation of permanent chemical cross‐links, imposing additional geometric constraints on chain dynamics, confers to the rubber improved mechanical strength, chemical and thermal stability, elasticity, and elastic recovery from deformation, and durability, as well as a good balance between the different viscoelastic properties. It is therefore clear how the study of the cross‐linking density and that, more detailed, of the specific motions affected by cross‐linking are of great interest to better understand the relationship between cross‐linking and the macroscopic properties it confers to the rubbers.

NMR is a natural candidate to investigate these aspects. Indeed, NMR techniques have often been used to get information on cross‐linking, typically through the measurement of ^1^H *T*_2_ relaxation times or by application of DQ NMR techniques, which allow the measurement of the ^1^H‐^1^H residual dipolar coupling (*D*_res_) left unaveraged by the nonisotropic motions of the polymer chains [67, 95, 96, 97]. On the other hand, ^1^H FC NMR has been employed to investigate the effect of cross‐linking on both segmental and collective polymer dynamics.

A summary of the rubber samples investigated by ^1^H FC NMR relaxometry is reported in Table 3, together with vulcanization conditions. In the early 2000s, Kariyo and Stapf [63, 64, 72] reported the influence of cross‐linking on the NMRD curves of NR and BR obtained by vulcanization at different sulfur and accelerator contents with same sulfur/accelerator ratio. At the same time, NR cross‐linked with fixed sulfur/accelerator ratio and different sulfur contents were also studied by Chaumette et al. [68] More recently, Martini et al. [66] studied the effect on the relaxation behavior of IR, BR, and SBR induced by cross‐link density and length of sulfur bridges on samples prepared by varying the sulfur/accelerator ratio and vulcanization temperature. Similar studies were carried out by Nardelli et al. [67, 70] on IR and NR vulcanized at different sulfur/accelerator ratio and vulcanization temperature. In all these studies, ^1^H FC NMR experiments were performed by commercial relaxometers, allowing *R*_1_ to be measured as a function of Larmor frequency down to 10 kHz. Only very recently, Rössler and coworkers [69] were able to perform experiments at very low frequencies (down to 500 Hz) on BR and NR, profiting from a home‐made relaxometer.

**TABLE 3:** Composition and curing temperature of the rubber samples investigated by ^1^H FC NMR relaxometry to unravel the effect of cross‐linking on dynamics.

Polymer	Cure package	Curing temperature	Ref.
BR	ZnO 3 phr Stearic acid 2 phr TBBS 1‐4 phr Sulfur 1‐4 phr Sulfur/TBBS = 1	433 K	Kariyo and Stapf [72] Stapf and Kariyo [98]
	ZnO 3 phr Stearic acid 2 phr TBBS 3 phr Sulfur 1‐3 phr	423 K	Martini et al. [66]
	ZnO 5 phr Stearic acid 2 phr CBS 0.14‐1.4 phr Sulfur 0.7‐7 phr Sulfur/CBS = 5	433 K	Becher et al. [69]
IR	ZnO 3 phr Stearic acid 2 phr TBBS 3 phr Sulfur 1‐3 phr	423 K	Martini et al. [66]
	ZnO 3 phr Stearic acid 2 phr TBBS 3 phr Sulfur 1 phr	443 K	Martini et al. [66]
	ZnO 3 phr Stearic acid 2 phr TBBS 2 phr Sulfur 2 phr	443 K	Martini et al. [66]
	ZnO 3 phr Stearic acid 2 phr TBBS 1 phr Sulfur 3 phr	443 K	Martini et al. [66]
	ZnO 3 phr Stearic acid 2 phr TBBS 3 phr Sulfur 2 phr	423 K	Nardelli et al. [70]
	ZnO 3 phr Stearic acid 2 phr TBBS 3 phr Sulfur 1‐3 phr	423, 433, 443 K	Nardelli et al. [67]
NR	ZnO 3 phr Stearic acid 2 phr TBBS 1‐7 phr Sulfur 1‐7 phr Sulfur/TBBS = 1	433 K	Kariyo and Stapf [72] Kariyo and Stapf [63] Kariyo and Stapf [64]
	CBS 1‐5 phr Sulfur 1‐5 phr Sulfur/CBS = 1	—	Chaumette et al. [68]
	ZnO 3 phr Stearic acid 2 phr TBBS 3 phr Sulfur 1‐3 phr	423, 433, 443 K	Nardelli et al. [67]
	ZnO 5 phr Stearic acid 2 phr CBS phr 0.14‐1.4 Sulfur 0.7‐7 phr Sulfur/CBS = 5	433 K	Becher et al. [69]
	Dicumyl peroxide 1 and 4 phr	433 K	Becher et al. [69]
SBR	ZnO 3 phr Stearic acid 2 phr TBBS 3 phr Sulfur 1‐3 phr	423 K	Martini et al. [66]

*Note:* TBBS and CBS stand for N‐tert‐butyl‐2‐benzothiazole sulfenamide and N‐cyclohexyl‐2‐benzothiazole sulfenamide accelerators, respectively.

**FIGURE 7:**
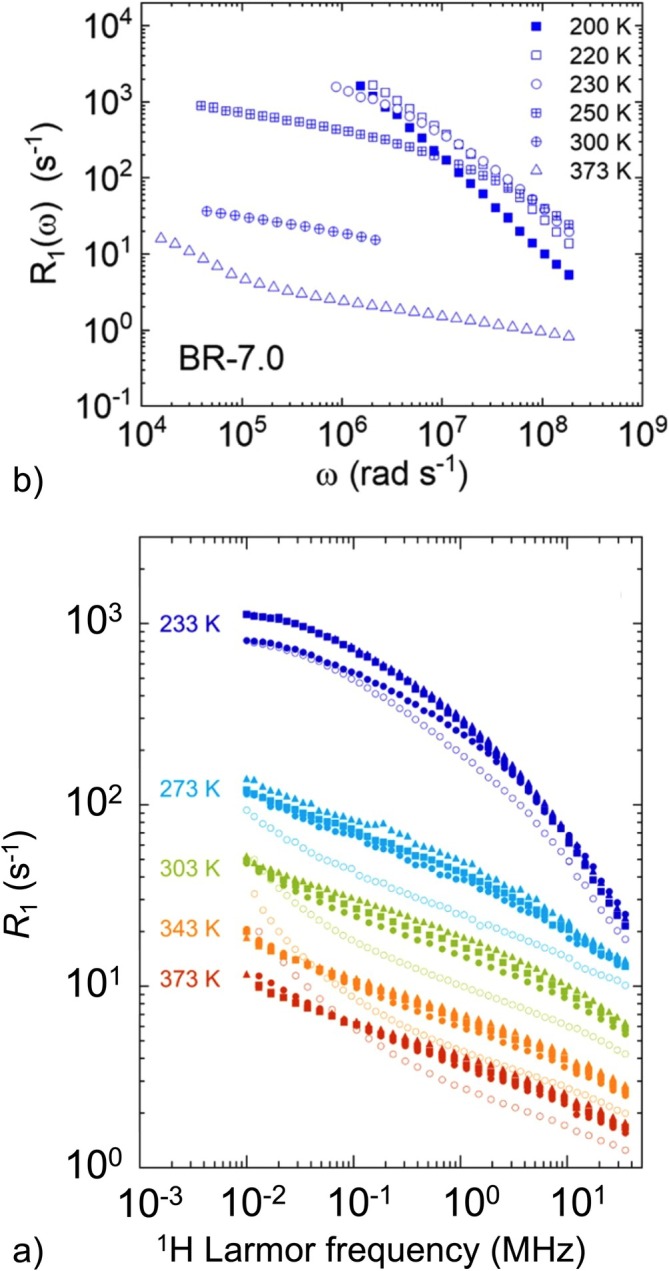
(a) ^1^H NMRD curves (10 kHz to 35 MHz) at the indicated temperatures of PB (empty circles) and BR samples at increasing degree of cross‐linking obtained by vulcanization with increasing sulfur content: 1 phr (full circles), 2 phr (full squares), and 3 phr (full triangles). Adapted with permission from Martini et al. [66] Copyright 2020 (ACS). (b) ^1^H NMRD curves (500 Hz to 40 MHz) at the indicated temperatures of a cross‐linked BR sample. Adapted with permission from Becher et al. [69] Copyright 2025 (ACS).

By comparing the NMRD curves of vulcanized and uncured elastomers, it was found that cross‐linking does not affect the slope of *R*_1_(*ω*) dispersions, keeping the same values of the *γ* exponent in the observed regimes (Figure 7a). On the other hand, a slowdown of both segmental and collective motions occurs with increasing cross‐link density, with a consequent shift of the NMRD curves toward lower frequencies, similar to that observed by lowering the temperature (Figure 7a,b). This behavior is related to the increase of the glass transition temperature upon cross‐linking.

Different regimes of polymer dynamics were observed depending on the type of polymer and experimental conditions. With commercial relaxometers and temperatures up to 373 K, only regime I of TR model (Rouse dynamics) was detected for all rubbers at all the investigated cross‐link densities. The use of a home‐made relaxometer allowed also regime II (constrained Rouse) to be revealed for BR and NR by Becher et al. [69] thanks to the lower achievable low‐frequency limit. This regime could be explored over a broader frequency range for BR thanks to the lower glass transition temperature. Interestingly, Chaumette et al. [68, 99] reported similar power law behavior and dependence on cross‐linking also for ^1^H longitudinal relaxation rates in the rotating frame (*R*_1*ρ*_) measured at different spin‐lock frequencies.

**FIGURE 8:**
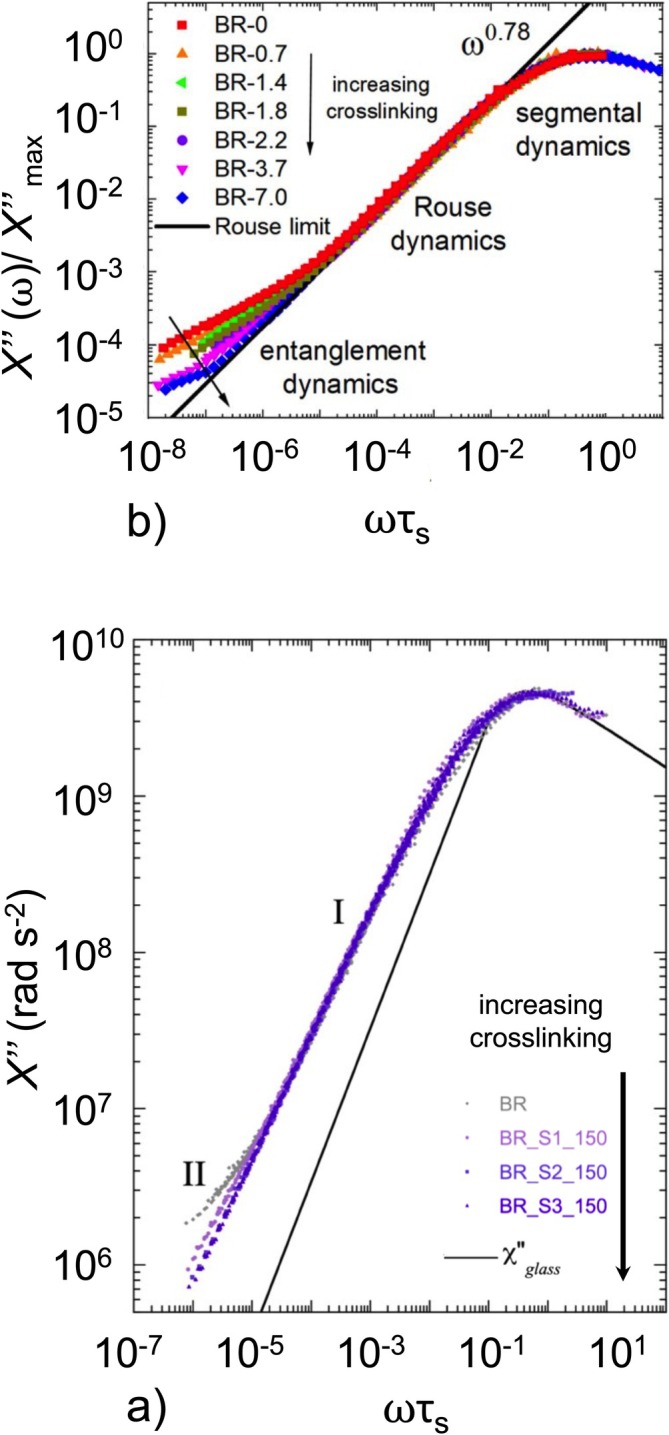
$\chi^{\prime \prime }\left(\omega \tau_{s}\right)$ master curves of BR samples with different cross‐linking degrees built using ^1^H FC NMR data acquired with (a) a commercial FC NMR relaxometer (10 kHz to 35 MHz) and (b) a home‐made relaxometer (500 Hz to 40 MHz). (a) Adapted with permission from Martini et al. [66] Copyright 2020 (ACS). (b) Adapted with permission from Becher et al. [69] Copyright 2025 (ACS).

In analogy to linear polymers, some authors used the NMR susceptibility representation and built $\chi^{\prime \prime }\left(\omega \tau_{s}\right)$ master curves exploiting the FTS principle to analyze data and determine $\tau_{s}$ values [66, 67, 69, 70]. The $\chi^{"}\left(\omega \tau_{s}\right)$ master curves obtained for BR samples by Martini et al. [66] at different cross‐link densities are shown in Figure 8a. The susceptibility spectra of all samples are superimposed down to *ωτ*_s_ ≃ 10^‐5^, covering regimes 0 and I, whereas the onset of regime II is clearly more and more protracted as cross‐link density increases. This trend was better highlighted by Becher et al. [69] for BR and NR thanks to the extension of FC data to lower frequencies (Figure 8b).

**FIGURE 9:**
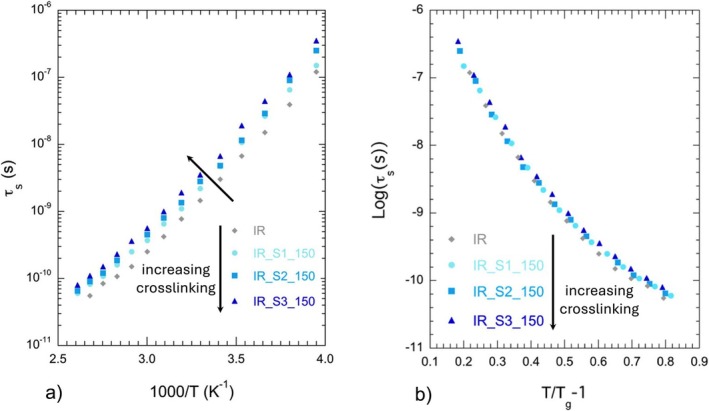
(a) Values of *τ*_s_ vs 1000/*T* and (b) of Log*τ*_s_ *vs* (*T*/*T*_g_ − 1) for IR samples with different degree of cross‐linking. Adapted with permission from Martini et al. [66] Copyright 2020 (ACS).

The construction of the master curves also enabled the determination of the correlation times for segmental motions, *τ*_s_, as a function of temperature, therefore quantifying the slowdown of these motions caused by cross‐linking [66, 69, 70]. As exemplified in Figure 9a for IR, the trends with temperature are very similar for samples at different cross‐linking degrees, but *τ*_s_ progressively increases at each temperature with increasing cross‐linking. Moreover, Martini et al. [66] reported a slight increase of *τ*_s_ for samples with a similar cross‐link degree, but a higher fraction of polysulphidic links, indicating that also the cross‐link type affects segmental dynamics. These findings were in agreement with the trends of *T*_g_ determined by differential scanning calorimetry (DSC). Indeed, by reporting Log*τ*_s_ *vs* (*T*/*T*_g_–1) (Figure 9b), a very good superposition of all the curves was observed, confirming the connection between the increase of *τ*_s_ and *T*_g_, without effects of cross‐linking on polymer fragility (see Section 2).

In the search for methods able to quantify the cross‐link density of rubbers (1/*M*_c_, where *M*_c_ is the average molar mass between two adjacent cross‐links) alternative to equilibrium swelling [100], Nardelli et al. [67] tested the correlation between *R*_1_ at a given frequency in the Rouse regime and 1/*M*_c_ for a series of IR and NR samples. Indeed, the tire industry is looking for faster, solvent‐free, and model‐independent approaches to determine this fundamental parameter. The analysis showed a linear dependence of *R*_1_ on 1/*M*_c_ with different slopes for samples vulcanized at different temperatures, indicating that *R*_1_ values are also affected by the chemical modifications of the polymer chains occurring during vulcanization. Therefore, this method was judged less suitable for a direct determination of cross‐link density compared to other established NMR methods, such as ^1^H DQ NMR for the measurement of *D*_res_ and low‐field CPMG experiments for the measurement of ^1^H *T*_2_ [67, 96, 97].

## 5 Effects of Fillers and Additives on Dynamics

Fillers and additives are commonly mixed with elastomers in rubber production to improve processability and tailor thermal, mechanical, and rheological properties for specific applications [2].

The most important reinforcing filler is carbon black (CB), consisting of small spherical particles made up of graphitic layers of carbon with minor amounts of oxygen‐ and hydrogen‐containing functional groups located on the surface, obtained by combustion or thermal degradation of natural gas, light, and heavy crude oils and aromatic hydrocarbons under controlled oxygen conditions. Another commonly used reinforcing filler is finely divided silica, whereas kaolin and calcium carbonate are nonreinforcing fillers that only increase viscosity of the compound. Recently, the use of sustainable fillers, such as lignin, cellulose and silica derived from agricultural waste, has become an important area of research aiming to improve mechanical properties while reducing the environmental impact of rubber products [101, 102].

The reinforcement effect of fillers depends on characteristics such as morphology, surface area, and composition, as well as on polymer structure and filler–polymer interactions. The main molecular mechanisms at the basis of reinforcement of rubbers have been widely investigated and comprehend the hydrodynamic effect, the elastic properties of polymer network vulcanized in the presence of the filler, polymer−filler chemical and physical interactions at the interface, and filler−filler interactions [103, 104, 105]. For a filler to be reinforcing, it is important that particles have diameters of 10‐100 nm and that the elastomers adhere to them.

Polymer chains near filler surface exhibit altered dynamics compared to bulk chains, with dynamics varying with distance from the surface, temperature, and interaction strength. Attractive or repulsive interactions slow down or accelerate chain motion, producing a gradient of dynamics from the interface to the bulk. The presence of interfacial layers can also significantly influence the composites' properties such as glass transition, elasticity, and rheology. Despite decades of investigation, some of these aspects remain debated [106, 107, 108].

Time‐domain NMR has demonstrated to be one of the most useful tools to investigate the filler–rubber interactions by measuring the effect of the additional constraints imposed by absorption or proximity to fillers on structural and dynamic properties of the elastomer [109, 110, 111, 112, 113]. Even if the most used methods are the analysis of ^1^H FIDs [111, 114, 115] and DQ NMR [95, 116], the effect of filler particles on polymer dynamics has also been investigated by ^1^H FC NMR relaxometry. The samples studied and their compositions are summarized in Table 4.

**TABLE 4:** Composition and curing temperature of the rubber samples investigated by ^1^H FC NMR relaxometry to unravel the effect of fillers and resins on dynamics.

Polymer	Cure package	Curing temperature	Additives	Ref.
NR	ZnO 3 phr Stearic acid 2 phr TBBS 1phr Sulfur 1 phr	333 K	CB (N220) 20 and 50 phr	Kariyo and Stapf [72] Kariyo and Stapf [64]
IR	ZnO 3 phr Stearic acid 2 phr TBBS 3 phr Sulfur 2 phr	323 K	CB (N330) 80 phr	Nardelli et al. [70]
	ZnO 3 phr Stearic acid 2 phr TBBS 3 phr Sulfur 2 phr	323 K	Silica (Ultrasil 7000) 50 phr	Nardelli et al. [70]
	ZnO 3 phr Stearic acid 2 phr TBBS 3 phr Sulfur 2 phr	323 K	Silica (Ultrasil 7000) 15 phr CB (N330) 20 phr	Nardelli et al. [70]
BR	ZnO 3 phr Stearic acid 2 phr TBBS 1 phr Sulfur 1 phr	333 K	CB (N660) 10 and 30 phr	Kariyo and Stapf [72]
SBR	ZnO 3.5 phr Stearic acid 2 phr CBS 2 phr Sulfur 2 phr	443 K	TDAE 37.5 phr CB (N100) 45 phr Kristalex 5140 0 and 15 phr	Pierigé et al. [71, 73]
	ZnO 3.5 phr Stearic acid 2 phr CBS 2 phr Sulfur 2 phr	443 K	TDAE 37.5 phr CB (N100) 45 phr SMD‐31144 15 phr	Pierigé et al. [73]
	ZnO 3.5 phr Stearic acid 2 phr CBS 2 phr Sulfur 2 phr	443 K	TDAE 37.5 phr CB (N100) 45 phr Dertoline MG 15 phr	Pierigé et al. [73]
	ZnO 3.5 phr Stearic acid 2 phr CBS 2 phr Sulfur 2 phr	443 K	TDAE 37.5 phr CB (N100) 45 phr Kristalex 5140 15, 25, 35, and 45 phr	Pierigé [117]
	ZnO 3.5 phr Stearic acid 2 phr CBS 2 phr Sulfur 2 phr	443 K	TDAE 37.5 phr CB (N100) 45 phr SMD‐31144 15, 25, 35, and 45 phr	Pierigé [117]
	ZnO 3.5 phr Stearic acid 2 phr CBS 2 phr Sulfur 2 phr	443 K	TDAE 37.5 phr CB (N100) 45 phr Dertoline MG 15, 25, 35, and 45 phr	Pierigé [117]

*Note:* TDAE stands for treated distilled aromatic extract used as plasticizer.

**FIGURE 10:**
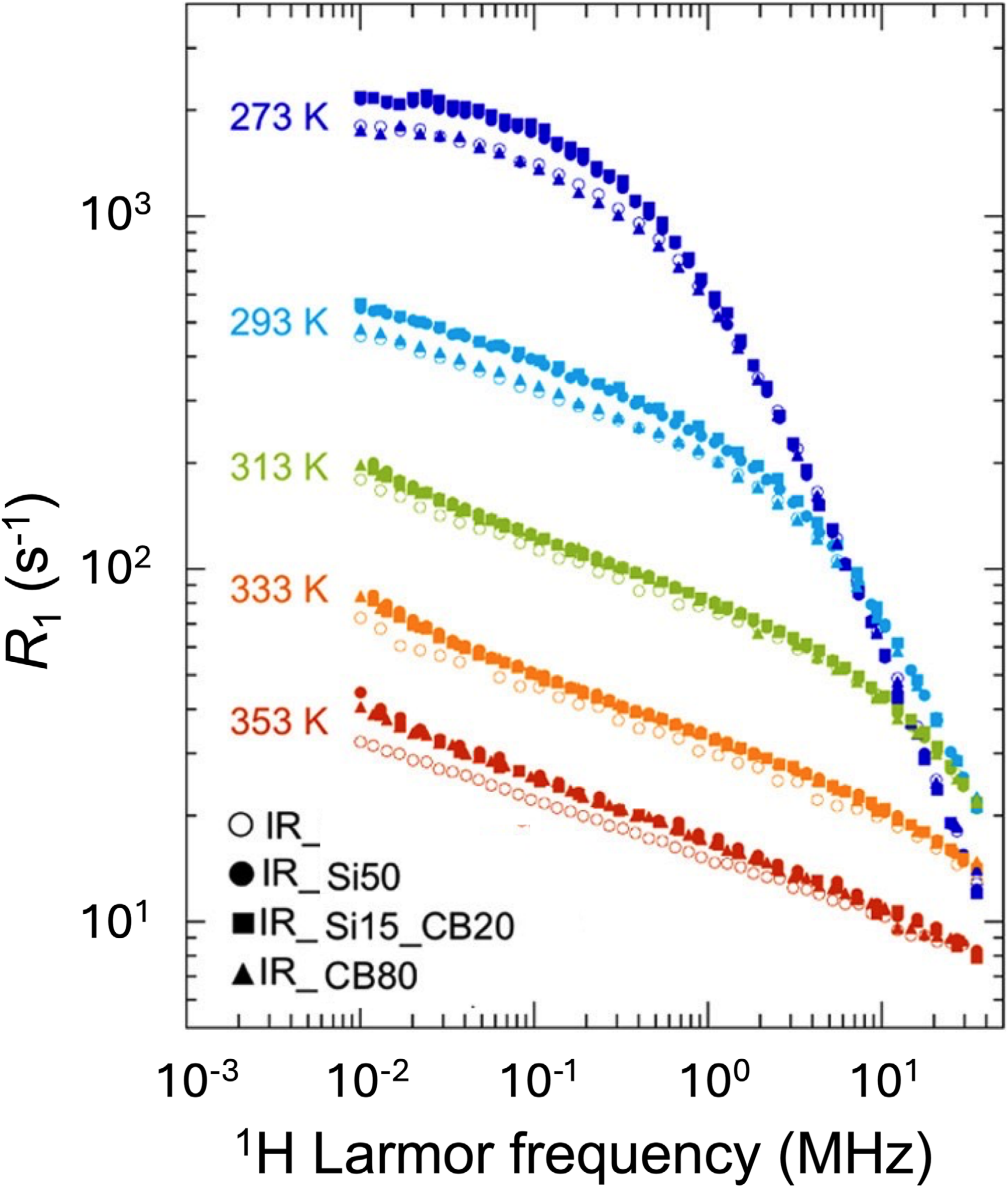
*R*_1_(*ω*) dispersion curves of IR and IR filled with silica (IR_Si50), carbon black (IR_CB80) and both fillers (IR_Si15_CB20) at different temperatures. Reprinted with permission from Nardelli et al. [70] Copyright 2021 (ACS).

**FIGURE 11:**
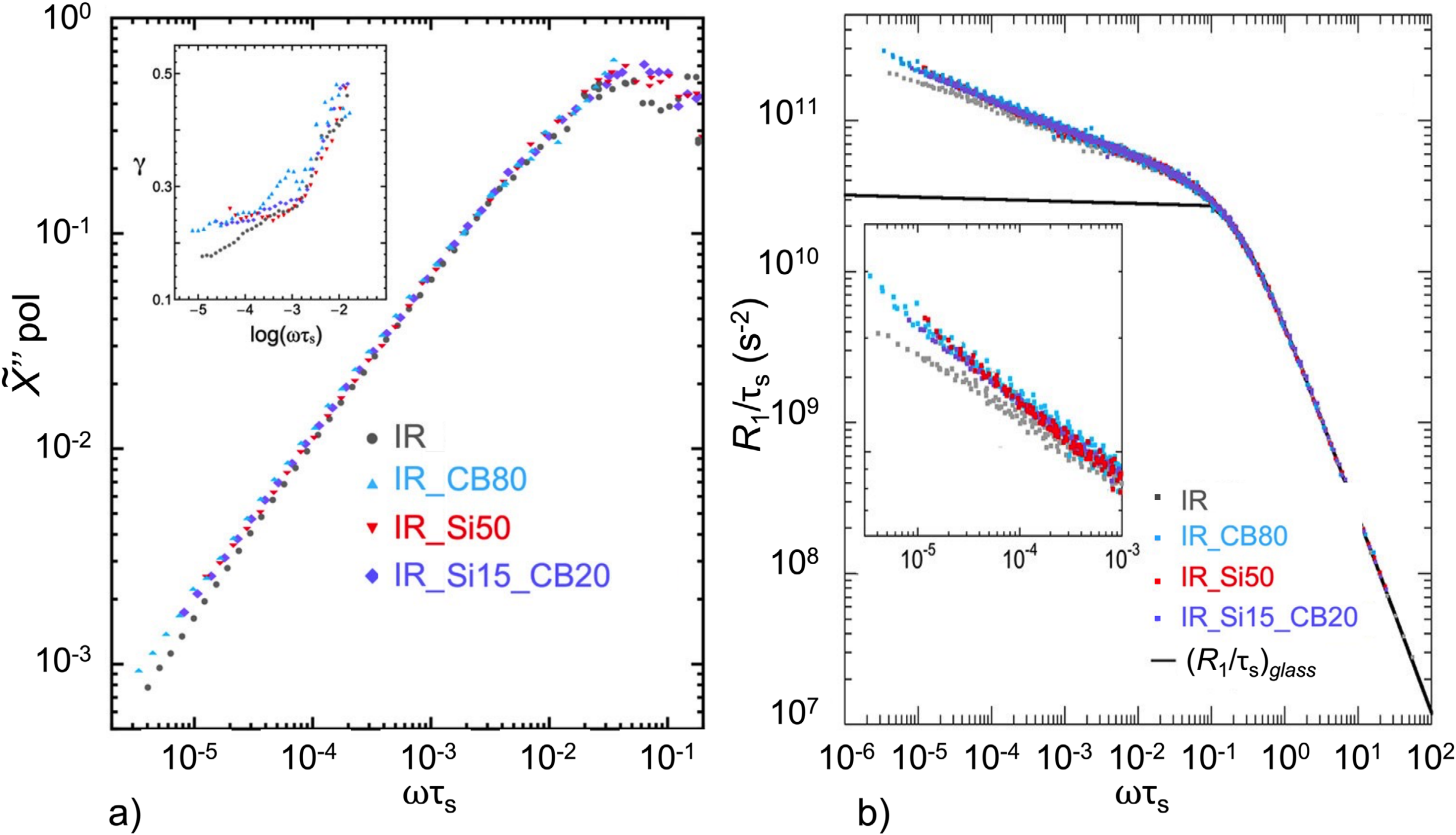
(a) $\chi_{\mathrm{pol}}^{\prime \prime }\left(\omega \tau_{s}\right)$ spectra and (b) $R_{1}\left(\omega \tau_{s}\right)/\tau_{s}$ master curves of IR and IR filled with silica (IR_Si50), carbon black (IR_CB80) and both fillers (IR_Si15_CB20). In the inset of panel (a) the values of the *γ* exponent are reported. Reprinted with permission from Nardelli et al. [70] Copyright 2021 (ACS).

Kariyo and Stapf [64] studied NR loaded with two different contents (20 and 50 phr) of CB (N220) and vulcanized in the same conditions by recording ^1^H NMRD curves. No differences were observed at room temperature with respect to the unfilled rubber, but small increases in *R*_1_ values (maximum 12%) were found at low frequencies for the curves recorded at high temperatures (above 313 K). A similar behavior was observed for BR loaded with 10 and 30 phr of CB (N660) [72]. More recently, Nardelli et al. [70] investigated the effect of loading CB (N330) and silica (Ultrasil 7000), either alone or combined, on IR (Table 4). For all samples, an increase in *R*_1_ was observed at high temperature in the low frequency region with respect to the unfilled IR (Figure 10). These differences were better highlighted by separating the contributions of polymer dynamics ( $\chi_{\mathrm{pol}}^{\prime \prime }\left(\omega \tau_{s}\right)$) by subtracting that of segmental dynamics ( $\chi_{\text{glass}}^{\prime \prime }\left(\omega \tau_{s}\right)$) from the total $\chi^{\prime \prime }\left(\omega \tau_{s}\right)$ spectrum, as discussed in Section 2, and passing to the $R_{1}\left(\omega \tau_{s}\right)/\tau_{s}$ representation (Figure 11). The obtained $\chi_{\mathrm{pol}}^{\prime \prime }\left(\omega \tau_{s}\right)$ spectra (Figure 11a) were found to be affected by the presence of fillers in the Rouse regime, where different power law dependences on reduced frequency were found for filled IR samples with respect to the unfilled one. As shown in the inset of Figure 11a, at low reduced frequencies (*ωτ*_s_ ≤ 10^‐3^), *γ* ranges between 0.22 and 0.26 for the filled samples, while it is ∽0.18 for unfilled IR. Correspondingly, changes in the frequency dependence of $R_{1}\left(\omega \tau_{s}\right)/\tau_{s}$ at low reduced frequencies were observed for all filled rubbers (Figure 11b). These findings have been associated with changes in chain conformations induced by interactions with surfaces, resulting in partial chain alignment close to the surface, and in different mode distributions at the length scale of Rouse motions. On the other hand, the dependences of *τ*_s_ values on temperature, as determined from the construction of the $\chi^{\prime \prime }\left(\omega \tau_{s}\right)$ master curves, indicated that segmental dynamics is only slightly modified by the presence of fillers.

Resins are key additives of tire elastomer compounds, which are employed as tackifying, curing, or reinforcing agents. From the chemical perspective, resins used in the tire industry are oligomeric compounds based on hydrocarbons, phenol‐formaldehyde polymers, or rosins, characterized by high softening points and glass transition temperatures [118]. Therefore, they typically induce an increase of the *T*_g_ of the rubber compound. Tackifying resins are introduced to favor the processability of the uncured compound, to promote filler dispersion, and to optimize the so‐called “magic triangle” of tire performance, namely rolling resistance, wet grip, and tread life. Their role and the properties provided to the final product depend on how the resin alters the chain dynamics of the polymeric matrix, which in turn is connected to resin physicochemical characteristics, including chemical structure, molecular weight, softening point and *T*_g_, as well as to resin concentration and polymer–resin miscibility [119, 120, 121, 122, 123].

**FIGURE 12:**
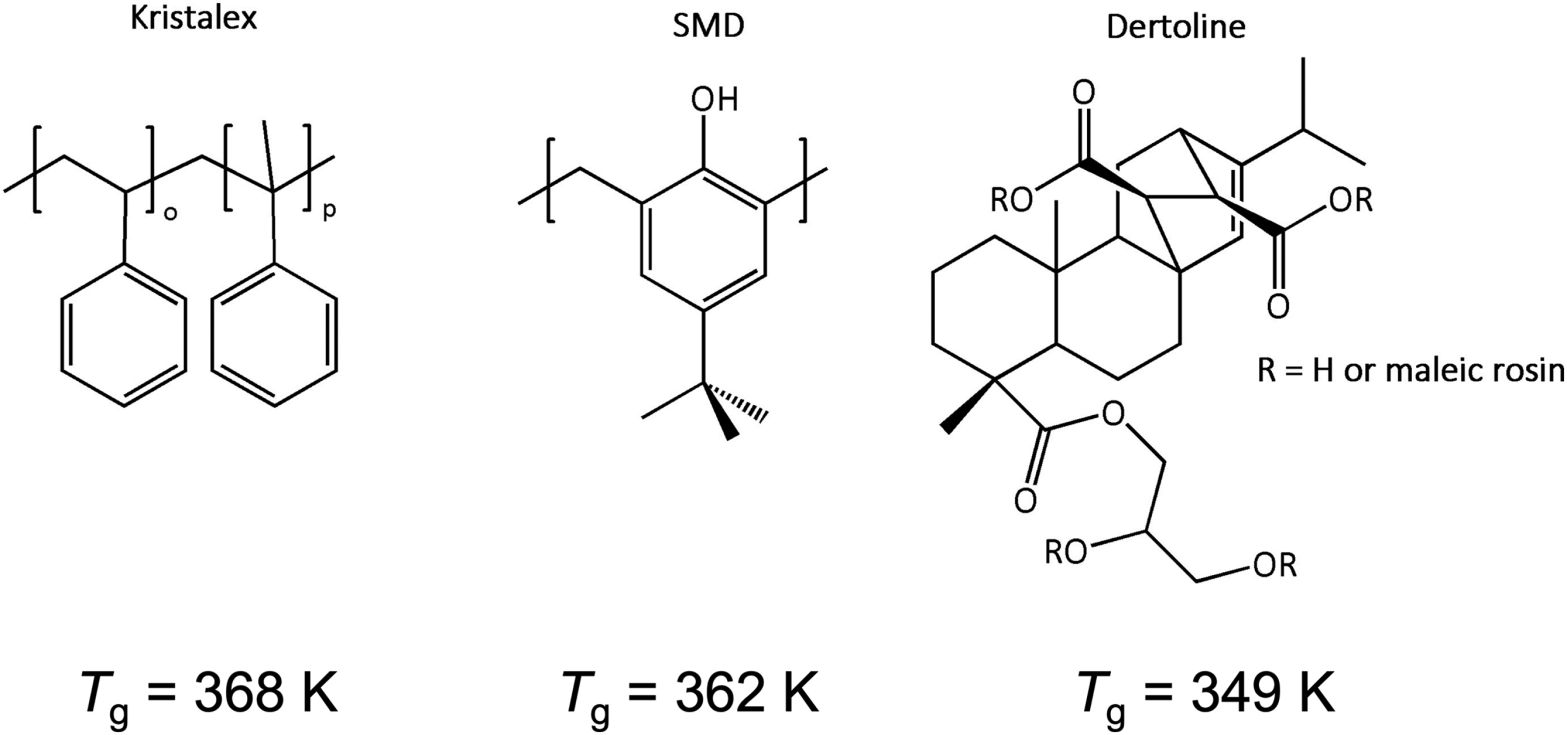
Chemical structures and glass transition temperatures (*T*_g_) of Kristalex 5140 (Kristalex), SMD‐31144 (SMD), and Dertoline MG (Dertoline) resins.

Recently, ^1^H FC NMR relaxometry has been employed to probe how different types of tackifying resins, bio‐based or synthetic (Figure 12), affect polymer chain dynamics in tire model compounds based on SBR (Table 4). Specifically, in two multitechnique studies by Pierigé et al. [71, 73], ^1^H FC NMR was combined with other NMR spectroscopy and relaxometry methods to assess the influence of different resins on both the structural and dynamic features of uncured and cured SBR compounds. In the first study [71], variable temperature NMRD curves were recorded on SBR compounds with and without 15 phr of Kristalex resin (Figure 12). The subsequent work [73] extended the analysis to the SMD and the bio‐based Dertoline resin (Figure 12) at the same concentration.

**FIGURE 13:**
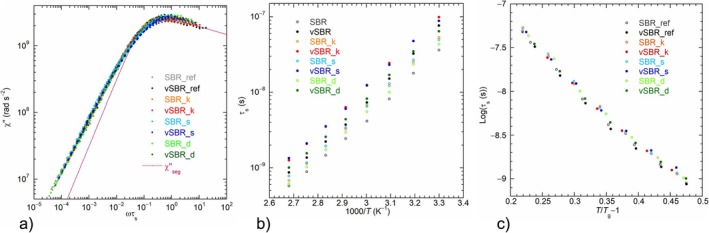
(a) $\chi^{\prime \prime }\left(\omega \tau_{s}\right)$ master curves obtained for the investigated samples. The contribution of the sole segmental dynamics ( $\chi_{\mathrm{seg}}^{\prime \prime }\left(\omega \tau_{s}\right)$), calculated on the basis of the Cole–Davidson spectral density function, is also shown (red line). (b) Correlation times for segmental dynamics (*τ*_s_) as a function of inverse temperature (1000/*T*). (c) Log*τ*_s_ *vs* (*T*/*T*_g_ − 1). Reprinted from Pierigé et al. [73].

For all samples, a single ^1^H *R*_1_ value was measured at all temperatures and frequencies. This result indicates an intimate mixing between resin and elastomer, allowing a complete averaging of relaxation rates by spin diffusion. Moreover, it was found that the addition of resin does not affect the NMRD profiles, apart from a shift of the curves toward lower frequencies consistent with a slowdown of segmental dynamics. This behavior was reflected in an increase of *τ*_s_, obtained by the construction of the NMR susceptibility master curves (Figure 13), upon resin addition. Interestingly, *τ*_s_ increased with different extents depending on the specific resin, with the following order: Dertoline < SMD < Kristalex (Figure 13b), in agreement with the trend of resin *T*_g_ values. When τ_s_ was plotted against the reduced variable *T*/*T*_g_–1, the curves of all samples collapsed onto the same master curve, indicating that resin addition has a little impact on the overall fragility of the polymer matrix under the investigated conditions.

The influence of resin concentration was further investigated by Pierigé [117] through the study of SBR samples with increasing content (from 15 to 45 phr) of Kristalex or Dertoline resin. Even at the highest resin content, a single *R*_1_ was measured at each temperature and frequency. For uncured samples, *τ*_s_ progressively increased with resin content, reflecting a progressive slowdown of segmental dynamics. This trend was not retained in the case of cured samples, since the increase of resin contents also caused a decrease in cross‐link density, thus resulting in two opposing effects on segmental dynamics. In all cases, the resin did not affect polymer fragility.

## 6 Conclusions

Applications of FC NMR relaxometry to the investigation of ^1^H longitudinal relaxation on elastomers and rubbers of interest for the tire industry over the last two decades have demonstrated the potential of this technique in disclosing the dynamic properties of these systems in dependence on polymer length, cross‐link density, vulcanization conditions, and presence of fillers and additives. By performing relaxation rate measurements over quite broad Larmor frequency ranges as a function of temperature and exploiting the FTS principle, information on dynamics was obtained over a wide range of frequencies encompassing segmental dynamics and entangled polymer dynamics. Segmental dynamics could be thoroughly characterized, determining the temperature dependence of motional correlation times. Relationships were established between correlation time values and chain length in the case of elastomer melts, whereas for vulcanized elastomer compounds correlation times were found to depend on rubber properties (i.e., cross‐link density and sulfur bridge nature) and composition (i.e., presence of fillers or tackifying resins). The use of home‐built relaxometers giving access to relaxation rates at Larmor frequencies on the order of 100 Hz was necessary to study polymer dynamics in the entanglement regime for both long chain and cross‐linked elastomers.

However, the application of FC NMR relaxometry to elastomers and rubbers still faces significant technical and theoretical challenges. Developments in the hardware and software of FC technology to move toward lower frequencies and shorter switching time values, as well as high‐temperature (> 373 K) measurements, required to investigate polymer dynamics in the entanglement regime in the kHz range and below, are needed on commercial relaxometers. Moreover, even when technical difficulties are overcome with the present state of the art home‐made instrumentation, the question remains of whether physical limits exist that restrict applications in these respects. In particular, measurements and interpretation at extremely low frequencies might become doubtful because of either the violation of the Redfield condition or local fields produced by secular spin interactions in systems with motional restrictions on time scales longer than *T*_1_. Moreover, FC NMR covers only a certain window of polymer dynamics and needs to be combined with other techniques, either NMR or rheology, dielectric spectroscopy, neutron scattering and so on, to achieve a comprehensive picture of polymer dynamics, as all these techniques afford limited and indirect information. Another challenge of FC NMR relaxometry in the investigation of elastomers and rubber dynamics concerns data interpretation, which requires several models and approximations to extract information on molecular motions starting from measured relaxation rates. Questions remain open on the interpretation of relaxation rate dispersions in terms of models for polymer dynamics. For non–cross‐linked elastomer melts, a detailed scenario has been established by FC NMR relaxometry, which can be interpreted in the frame of the TR model describing Rouse and entanglement dynamics. For rubbers, recent low‐frequency measurements revealed that highly cross‐linked networks ultimately feature only Rouse dynamics (truncated at the level of the network chain), while a second polymer relaxation regime with a power law exponent reflecting entanglement dynamics (constrained Rouse regime) is observed for lowly cross‐linked networks. However, the interpretation of power law exponents in terms of intramolecular and intermolecular contributions to relaxation and contributions from motionally heterogeneous subensembles of the network (polymer chains in the network, defects, short and long dangling chains) is not straightforward. Moreover, the separation of intramolecular and intermolecular contributions to relaxation requires laborious isotope dilution experiments, and it is impracticable for elastomer compounds or rubbers. The situation becomes even more complicated when real elastomer compounds are considered, where components, such as resins, oils, or fillers, with different dynamics may contribute to the overall relaxation rate, through spin diffusion between different components.

Although it cannot be yet considered a routine characterization tool in the tire industry, as it requires expert knowledge for the proper interpretation of data and continuous development and validation of theoretical models, FC NMR relaxometry remains a powerful experimental tool for the investigation of elastomers and rubbers dynamics, giving information inaccessible by other techniques.

## Author Contributions

**Francesca Nardelli:** conceptualization, writing – original draft, writing – review and editing, investigation, visualization. **Michele Pierigé:** writing – review and editing, visualization. **Elisa Carignani:** conceptualization, writing – original draft, writing – review and editing, investigation, visualization. **Marco Geppi:** conceptualization, writing – original draft, writing – review and editing, investigation, visualization. **Francesca Martini:** conceptualization, writing – original draft, writing – review and editing, investigation, visualization, supervision. **Lucia Calucci:** conceptualization, writing – original draft, writing – review and editing, investigation, visualization, supervision.

## Data Availability

Data sharing is not applicable to this article as no datasets were generated or analyzed during the current study.

## Appendix A

**TABLE A1:** List of symbols used in the theoretical background section.

Symbol	Definition
*T*_g_	Glass transition temperature
*τ*_s_	Correlation time of segmental motions
*τ*_α_	Correlation time of the α‐process
*M*_e_	Average molar mass between entanglements
$\left\langle r^{2}\left(t\right)\right\rangle$	Mean‐squared displacement (MSD)
*τ*_e_	Correlation time marking the limit between free Rouse and constrained Rouse regimes (Figure 2)
*τ*_R_	Correlation time marking the limit between constrained Rouse and reptation regimes (Figure 2)
*τ*_d_	Correlation time marking the limit between reptation and free diffusion regimes (Figure 2)
*C*_rot_(*t*)	Rotational correlation function
*C*_trans_(*t*)	Translational correlation function
*R*_1_ = 1/*T*_1_	Spin lattice relaxation rate
*J*(*ω*)	Spectral density function
$\chi^{"}\left(\omega \right)$	NMR susceptibility
*γ*	Exponent of the *R*_1_(*ω*)∝*ω* ^‐ *γ*^ power law
*α*	Exponent of the *C*(*t*)∝*t* ^*−α*^ power law
*R*_1,*intra*_	Contribution to *R*_1_ arising from intramolecular dipolar interactions
*R*_1,*inter*_	Contribution to *R*_1_ arising from intermolecular dipolar interactions
*J*_*CD*_(*ω*)	Cole–Davidson spectral density function
*β*_*CD*_	Cole–Davidson parameter (0 < *β*_*CD*_ ≤ 1)
*τ*_*CD*_	Cole–Davidson correlation time (*τ*_s_ = *β*_*CD*_ *τ*_*CD*_)
$C_{\mathrm{rot}}^{\mathrm{iso}}\left(t\right)$	Rotational correlation function in the so‐called “isotropic” models, such as renormalized Rouse
$C_{\mathrm{rot}}^{\mathrm{TR}}\left(t\right)$	Rotational correlation function in the Tube Reptation model
${\tilde{\chi }}^{"}\left(\omega \right)$	Normalized NMR susceptibility
$\chi {"}_{\mathrm{DD}}\left(\omega \right)=\omega R_{1}\left(\omega \right)$	Dipolar non‐normalized NMR susceptibility, also indicated as $\chi^{"}\left(\omega \right)$ in the manuscript
$\chi {"}_{\text{glass}}\left(\omega \right)$	Contribution of glassy (segmental) dynamics to $\chi^{"}\left(\omega \right)$
$\chi {"}_{\mathrm{pol}}\left(\omega \right)$	Contribution of polymer dynamics to $\chi^{"}\left(\omega \right)$
*f*	Fractional contribution of polymer dynamics to $\chi^{"}\left(\omega \right)$ (Equation (6))
*S*	Order parameter that represents a measure of the spatial restrictions imposed on segmental motions by chain connectivity and entanglements
*S*_*chain*_	Chain order parameter (Equation (7))
*τ*_0_	Pre‐exponential factor of VFT equation (Equation (9))
*T*_0_	Temperature at which the correlation time diverges to infinity in VFT equation (Equation (9))
*D*	Fragility factor in VFT equation (Equation (9))
*m*	Fragility index defined in Equation (10)
